# Precision Localization of Lipid‐Based Nanoparticles by Dual‐Fluorescent Labeling for Accurate and High‐Resolution Imaging in Living Cells

**DOI:** 10.1002/smsc.202300084

**Published:** 2023-06-27

**Authors:** Wen-Qiu Huang, Peter C. Burgers, Mohamadreza Amin, Theo M. Luider, Timo L. M. ten Hagen

**Affiliations:** ^1^ Precision Medicine in Oncology (PrMiO) Department of Pathology, and Nanomedicine Innovation Center Erasmus (NICE) Erasmus MC Cancer Institute 3015 GD Rotterdam The Netherlands; ^2^ Clinical and Cancer Proteomics Department of Neurology Erasmus MC 3015 GD Rotterdam The Netherlands

**Keywords:** high-resolution live cell imaging, lipid nanoparticles, lipophilic fluorescent labels, liposomes, precision nanomedicine

## Abstract

In nanomedicine, lipid‐based nanoparticles (NPs) such as liposomes (LPs) have established an important position. Precise delineation of NP interaction with cells and detailed characterization of activity are becoming essential, which mainly rely on labeling with lipophilic fluorescent molecules and assuming stable association with NPs. However, because of label separation from NPs in (biological) media, or when processed by cells, fluorescence‐based detection of an NP incorporating a single label may not necessarily indicate the actual presence of an NP but may be from the dissociated label, rendering results unreliable. Herein, flow cytometry and confocal microscopy are employed to demonstrate that to verify the localization of LPs in a cell with perfect accuracy, dual‐labeling, and contemporaneous detection of both fluorescent signals in one pixel are required. This is combined with size exclusion chromatography (SEC) and mass spectrometry measurements to indicate factors involved in label dissociation, which helps to understand the possible conditions of dissociated label and NP. It is shown that determining label colocalization with, and label dissociation from, dual‐labeled NPs are needed to provide accurate spatiotemporal insight into targeting destination (colocalized signals) and disintegration (separated signals) of NPs during intracellular processing and in studying payload delivery with precision in nanomedicine.

## Graphical abstract

Liposomes are one of the promising carriers for cancer theranostics, but the labeling instability of a carrier renders intensity‐based evaluation of nanoparticles (NP) cellular uptake unreliable. In this work, the potential risk of label dissociation in biological environment is investigated: the utilization of a dual labeling strategy for precise delineation of NPs especially by live cell imaging microscopy is advocated.

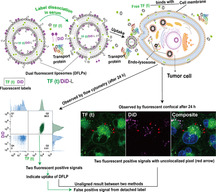

## 1 Introduction

Lipid‐based nanoparticles (NPs) such as liposomes (LPs) and lipid nanoparticles (LNPs) have emerged as promising carriers in the field of nanomedicine due to their amphipathic feature, which is useful for the transfer of drugs, antibodies, small molecules, and DNA and RNA for the purpose of chemotherapy,^[^ ^1^ ^]^ immunotherapy,^[^ ^2^ ^]^ and recently vaccines, as became obvious in the battle against COVID‐19.^[^ ^3^ ^]^ Researchers investigated numerous forms of LPs with varying stability for enhanced therapeutic efficacy, by modifying lipid composition, acyl chain length, lipid saturation, and surface charge of the head group.^[^ ^4^, ^5^ ^]^ Modification of these biochemical properties determines the fate of NPs and content delivery in targeted tissue or/and at specific compartments of a cell, and eventually determines efficient clinical translation and outcome in patients. Nevertheless, biological heterogeneity in diseases and patients hampers the clinical translation of these nano‐based strategies.^[^ ^6^ ^]^ To understand delivery and to optimize lipid‐based carriers for precision medicine, as well as personalized medicine, it is critical to determine precisely the destiny of NPs and contents inside target cells, which is mainly explored by the monolabeling of these carriers.

However, as accuracy concerns, few researchers^[^ ^7^, ^8^, ^9^ ^]^ have addressed the potential issue of labeling instability in carriers; labels commonly detach from carriers during incubation in media or when interacting with cells, which severely limits the reliability of intensity‐based systemic kinetics evaluation in vivo, and single‐molecule optical observation of NPs in tissues or cells. There is evidence that lipophilic probes can transfer instantly between NPs, from particles to cells and from cells to particles.^[^ ^7^, ^10^ ^]^ Furthermore, serum components such as lipoproteins,^[^ ^11^ ^]^ serum albumin,^[^ ^12^ ^]^ and other proteins^[^ ^13^, ^14^ ^]^ that serve as transporters for various compounds, including fatty acids, may influence labeling stability, and thus fluorescence, of NPs through lipid exchange and protein binding.^[^ ^15^, ^16^ ^]^ Nonetheless, in spite of validation of the integrity of fluorescent lipid markers in an ex vivo biological environment, once inside a cell, labeling instability may occur rendering unreliable results when studying intracellular behavior in detail. For instance, upon interaction of lipid‐based NPs with cells fluorescent markers may fuse to cell or organelle membranes during the uptake process,^[^ ^17^ ^]^ making it difficult to discern NP‐associated fluorescence from fluorescence coming from cell compartments after label‐dissociation. According to recent evidence, the dissociation and uptake of cyanine dyes can be attributed to the degradation of dye‐labeled nanostructures, which results in misleading positive intracellular fluorescence signal.^[^ ^18^ ^]^ Interestingly, Chong Qin et al.^[^ ^19^ ^]^ improved real‐time single particle level observation of individually dual‐color influenza viruses by incorporating quantum dots with distinct fluorescence. With this in mind, we examined the necessity of dual labeling of LPs for precise intracellular tracking. In this article, we give an insight into intrinsic elements that need to be considered when choosing fluorescent labeling. Furthermore, we used matrix‐assisted laser desorption ionization time‐of‐flight mass spectrometry (MALDI‐TOF MS) to evaluate the intricate reason of label separation from LPs under biological conditions.^[^ ^20^ ^]^

However, due to the complexity and multitude of interactions, which occur when fluorescent LPs (FLPs) are taken up by cells, we show that size exclusion chromatography (SEC), which is used to separate unbound labels and to evaluate the labeling stability of NPs in ex vivo condition,^[^ ^8^, ^21^ ^]^ is not sufficient, also flow cytometry data need to be regarded with care when single fluorochrome‐labeled NPs are used. Here, we argue that NPs need to be dual labeled to allow real intracellular delineation of NPs. Importantly, with flow cytometry we observed dye transition between two different cell groups incubated with distinct singly labeled FLPs (SFLPs). Moreover, we show by using dual FLPs (DFLPs) that intensity‐based cellular uptake evaluation of NPs by flow cytometry and confocal‐imaging‐based colocalization analysis do not align.

Taken together, the determination of localization of DFLPs in cells, where two fluorescent signals showed the highest correlation, underscore the significance of dual labeling for LPs, particularly in terms of boosting reliability and evaluating the efficiency of lipid‐based drug delivery system when conducting live cell super‐resolution microscopy. More so, next to guidance to the intracellular targeted destination of a carrier (when two fluorescent signals co‐localize), dual‐labeling may also provide a spatiotemporal indication of disintegration of the NP when processed by cells as well as payload release (when two fluorescent signals do not colocalize anymore). This has significance for the development and application of liposomes as well as other lipid‐based nanoparticles in personalized clinical settings.

## 2 Results and Discussion

### 2.1 Patterns of Fluorescent Label Detachment from LPs

**Figure 1:**
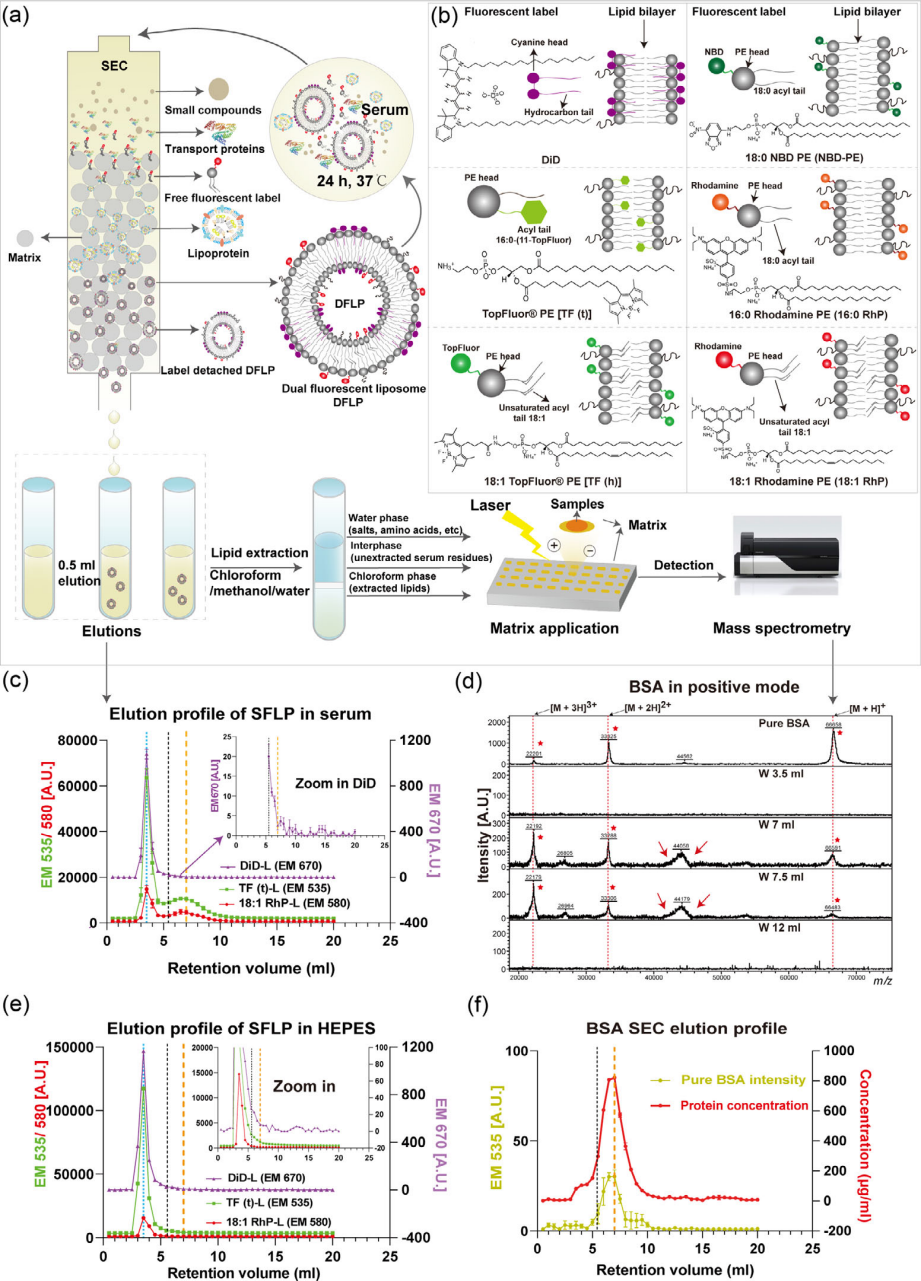
Dissociation of fluorescent liposome (FLP) in serum. a) Schematic process of size exclusion chromatography (SEC) and mass spectrum analysis of FLP incubated with serum. During SEC elution the largest elements (e.g., nanoparticles) depart the column first and the smallest elements (e.g., single small molecules) exit the column last. b) Schematic representation of chemical structures of fluorescent labels evaluated in this paper and locations in lipid bilayer. The fluorophore of each dye is colored, and the abbreviation of each fluorescent label is presented close to the commercial name. c) A typical elution profile for single FLP. The first peak, cutoff point and second peak are denoted in light blue, black and orange dashed lines, respectively. For better viewing the red and green elution curves are shifted 700 and 2000 points upwards, respectively. d) Mass spectral analysis of bovine serum albumin (BSA) in the positive ion mode using MALDI‐TOF‐MS. We extracted eluted fractions of 18:1 RhP labeled LP (18:1 RhP‐L) in serum and quantified the protein mass in the water phase. The BSA spectrum with three types of charges (the red dashed lines) is shown at *m/z* 66.6, 33.3, and 22.1 kDa, respectively, and the positive signals were marked with red stars. The red arrows point at a wide ranged peak near 44 kDa, likely containing a number of proteins. W3.5, W7, W7.5, and W12 denote the corresponding elution volumes extracted in the water layer. e) Representative elution profile of SFLPs in HEPES, the zoomed‐in image revealed that there was no peak at the orange dashed line. The cut‐point was shown by the black dashed line. The red and green curves are shifted 1000 and 3500 points for better viewing, respectively. f) Autofluorescence (left *y*‐axis) and protein concentration (right *y*‐axis) of eluted pure BSA.

We explored the dual‐labeling strategy with frequently used lipophilic fluorescent labels (**Figure** **1**b). Therefore, we combined the widely used carbocyanine dye DiD with fluorescent phospholipids TopFluor PE tail conjugated [TF (t)], TopFluor PE head group conjugated [TF (h)], NBD‐PE, Liss Rhodamine PE saturated (16:0 RhP), or unsaturated (18:1 RhP) to singly or dually label LP. For stability and quality assurance, fluorescent phospholipids were used at a concentration of 0.1 mol%, while DiD was used at a concentration of 0.005 mol% (Figure S1a, Supporting Information). We demonstrate that the insertion of two labels has no effect on the measured properties of the FLPs when kept in HEPES at 4 °C for up to 2 months (Figure S1b,c, Supporting Information).

We next performed SEC analysis to determine the dissociation of labels from the carrier (Figure 1a).^[^ ^22^ ^]^ After correction for autofluorescence coming from serum or unlabeled LP, the representative intensity‐based elution profiles of SFLPs (Figure 1c) and DFLPs (e.g., 18:1 RhP/DiD‐L) (Figure S2b, Supporting Information) showed two peaks at an elution volume of 3.5 mL (light‐blue dashed line) and 7 mL (orange dashed line), respectively, when NPs were incubated in serum. When FLPs were exposed to HEPES, irrespective of the label used, only a peak at 3.5 mL was observed and no second peak was identified (Figure 1e). Together with phosphorus assay of fractions collected during SEC (Figure S2c, Supporting Information, **2** d), the results indicate that the majority of FLPs were eluted in the first peak irrespective of buffer condition, in another word, when incubated in serum the fluorescent labels probably associate with serum components and were eluted in the second peak (Figure 1c). Importantly, DiD is associated rather in a stable way with LPs with no detectable second peak in serum in this ex vivo setting (Figure 1c).

**Figure 2:**
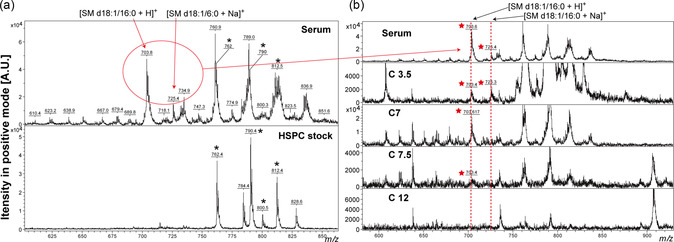
Mass spectrometry analysis of serum lipids in positive ion mode. a) Lipid content within serum was extracted and the lipid mass spectrum (first track), the two fragment ions at *m/z* 703.6 and 725.6 were identified as sphingomyelin (SM d18:1/16:0) [M + H]^+^ and [M + Na]^+^, which corresponds to the composition of LDL and HDL with in serum, respectively. The noise caused by the HSPC stock (a mixture of DSPC and DPPC, second track) are denoted with asterisks. b) Lipid content within the extracted elution fractions (18:1 RhP‐L in serum). The mass of SM (d18:1/16:0) was determined in serum as a positive control and representative results from eluted samples in corresponding volumes extracted in chloroform are referred to as “C3.5” “C7” “C7.5” and “C12”. The red dashed lines indicate the mass of fragment ions associated with HDL and LDL, while the red stars indicate positive signals.

### 2.2 MALDI‐TOF‐MS Reveals Transport Proteins Account for Extrinsic Elements Involved in Labeling Instability

#### 2.2.1 Proteins in Serum Contribute to the Dissociation of Fluorescent Labels

Interestingly, the autofluorescence of pure serum SEC eluent showed a peak at an elution volume of 7 mL (Figure S2a, Supporting Information, orange dashed line), which corresponds to the second peak of FLPs in serum (Figure 1c, S2b, Supporting Information, orange dashed line), we infer that dissociated labels interact with serum components that eluted at 7 mL. As albumin, a serum transport protein, is the main constituent of bovine serum^[^ ^23^ ^]^ and contains two tryptophan residues with intrinsic fluorescence,^[^ ^24^ ^]^ we hypothesized that bovine serum albumin (BSA) is present in the peak at 7 mL. Indeed, we observed comparable SEC profiles of pure BSA at 40 mg mL^−1^, the average concentration in serum, based on intensity (Figure 1f, gold curve, left *y*‐axis) and protein density (Figure 1f, red curve, right *y*‐axis).

To further support our observations and determine the involvement of albumin, we undertook MALDI‐TOF mass spectrometry (MS).^[^ ^25^ ^]^ The FLP 18:1 RhP‐L was incubated with serum for 24 h and evaluated by SEC (subsequently we used the same sample as a model for mass determination). From each eluted sample lipids were extracted in the chloroform phase by a previously published procedure (Figure 1a, S6, Supporting Information),^[^ ^23^ ^]^ and we found that BSA could be detected in the water phase in the positive ion mode. The mass spectral analysis of the singly charged species [M + H]^+^, doubly charged species [M + 2 H]^2+^, and triply charged species [M + 3 H]^3+^ of pure BSA was present at 66.6, 33.3, and 22.1 kDa, respectively (Figure 1d), which is consistent with earlier observations.^[^ ^26^ ^]^ Intriguingly, at this detection level, BSA fragment ions were absent in elution volumes 3–5 mL (fractions 6–10, 0.5 mL per fraction) and began to appear in volumes 5.5–10 mL (Figure 1d, S3a, Supporting Information), with volume of 7 to 8 mL exhibiting the highest abundance. Although this method cannot be used to determine quantitatively the amount of BSA involved in label dissociation, the mass distribution of BSA in elution volumes did match with the second peak of the SEC elution profile of FLPs (Figure S3b, Supporting Information). Electrospray ionization liquid chromatography mass spectrometry (ESI‐LC‐MS) verified the presence of BSA in these eluents, with a high peptide count in elution volume 7 and 7.5 mL, but a low count in elution volume 3.5 mL (Table S1, Supporting Information). The marginal coexistence of transport proteins with FLP in the first peak of the SEC profile is consistent with previous reports on the formation of the protein corona,^[^ ^15^, ^16^, ^27^ ^]^ which may attribute to the low protein (0.1%) binding properties of pegylated liposomes.^[^ ^28^, ^29^ ^]^ Interestingly, other proteins showed similar distribution with BSA, such as alpha‐fetoprotein (AFP) (Table S1, Supporting Information). These proteins have a comparable mass (69 kDa) as BSA and have been shown to have the ability to transport fatty acids and fluorescent lipid markers equal to BSA.^[^ ^14^ ^]^ In addition, the broad mass peak around 44 kDa (Figure 1d, S3a, Supporting Information, red arrow) had a similar SEC distribution compared to BSA and can most likely be attributed to serpin and fetuin as observed by proteomics (Table S1, Supporting Information). Both proteins have been shown to aid in storage and transport of a range of cargos in blood.^[^ ^13^, ^30^ ^]^ Finally, according to these data, serum proteins such as BSA, AFP, serpins, and fetuins most likely contributed to the detachment of fluorescent markers from LPs in serum.^[^ ^13^, ^15^, ^30^, ^31^ ^]^

#### 2.2.2 Lipoproteins are not the Specific Reason for Label Detachment

We need to note that defining all proteins in serum is not feasible due to limitations of our experiment setting, such as protein denaturation by the lipid extraction process, while this is also outside the scope of this article. However, lipoproteins, such as apolipoprotein A1 (ApoA1) and apolipoprotein E (ApoE), are recognized as one of the main components in the “protein corona” on liposome surfaces.^[^ ^28^ ^]^ The lipids regarding lipoproteins can be detected after extraction in chloroform by MALDI‐TOF MS in the positive mode based on their fatty acid chain composition and their head group. Due to the complexity of serum composition, we did not identify all of the peaks in the spectrum. However, we can assign masses to present between *m/z* 600 and 900 as fragment ions of high‐density lipoprotein (HDL) and low‐density lipoprotein (LDL).^[^ ^32^ ^]^ Such as *m/z* 703.6 and 725.6 (Table S2, Supporting Information, **Figure** **2**b, S4a, red dashed line) can be attributed as H^+^ and Na^+^ adducts of sphingomyelin (SM d18:1/16:0), respectively.^[^ ^32^, ^33^ ^]^ The size of HDL and LDL was reported as 5–15 and 18–28 nm, respectively,^[^ ^34^ ^]^ which will be primarily eluted after the void volume (Sepharose 4L‐4B pore size 35 nm) (Figure S4b, Supporting Information).^[^ ^35^, ^36^ ^]^ However, HDL‐ and LDL‐related lipid masses appeared in almost all eluents (3–11 mL) (Figure 2b, S4a, Supporting Information, red stars) instead of only in the volumes containing dissociated lipid markers (Figure S4b, Supporting Information, orange dashed line). We conclude that lipoproteins may play a role in label dissociation but are likely not that specific nor the main reason (supplemental discussion 1). Taken together, the mass spectrometry results uncovered the potential extrinsic reason for lipid marker detachment from FLPs, and we show that transport proteins in serum likely play an important role.

### 2.3 Trends in Fluorescent Label Dissociation Reveal the Importance of Intrinsic Aspects of Markers on Labeling Stability

**Figure 3:**
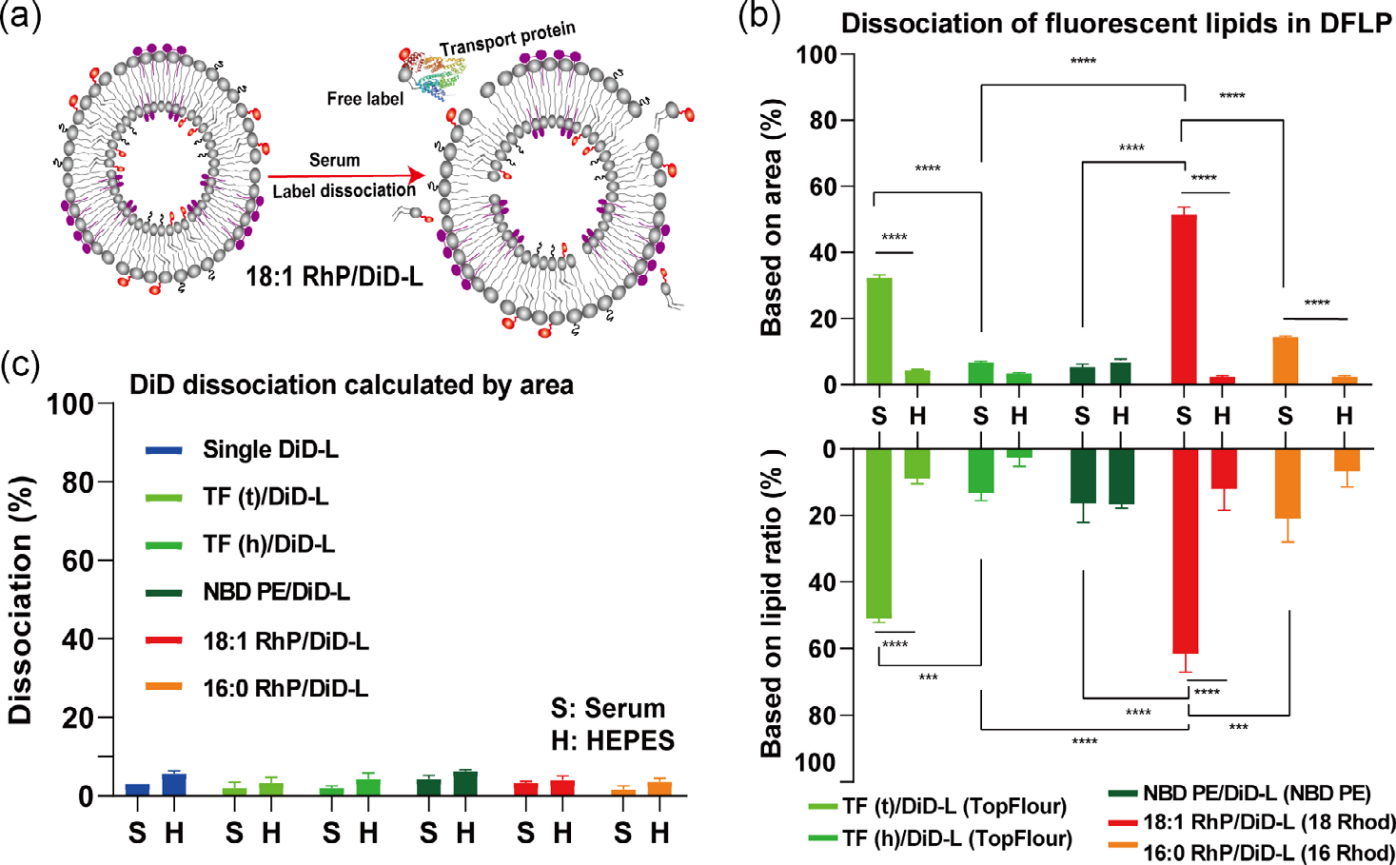
Dissociation percentage of labels from fluorescent liposomes (FLPs). a) Representative image of label detachment from dual FLPs (DFLPs) in serum. b) The dissociation percentage of each fluorescent lipid from DFLPs was determined using the intensity‐based area (positive *y*‐axis) and the label to lipid ratio‐based (negative *y*‐axis) analysis. c) Intensity‐based area evaluation of DiD dissociation from SFLPs and DFLPs. HEPES: H, Serum: S. Two‐way ANOVA with multiple comparisons was used to analyze these data. The error bars represent the standard error of the mean (S.E.M.), *n* = 3, ***p < 0.001, ****p < 0.0001 for comparisons within each subgroup.

To compare labeling stability, the proportion of area under the intensity‐based curve after 5.5 mL to the total area was calculated (Equation (1)). Detachment of DiD from SFLPs and DFLPs was relatively low (on average less than 6%) in both serum and HEPES, with no significant differences between these two groups (**Figure** **3**c), indeed, DiD was demonstrated to be stable in NPs when properly anchored.^[^ ^37^, ^38^ ^]^ However, a slightly reduced labeling stability was observed in HEPES compared to serum, which may be explained by self‐aggregation and changes in orientation in aqueous conditions, resulting in unstable anchoring of the long stearyl chain in the bilayer.^[^ ^39^ ^]^ Besides DiD, the stability of other fluorescent lipids in DFLPs was also determined; all dissociation of labels had a reasonably low value (on average below 6%) and did not differ significantly in the absence of serum (Figure 3b, positive *y*‐axis). However, in the presence of serum, these fluorescent phospholipids, with the exception of TF (h) and NBD‐PE, showed a significantly different degree of dissociation (on average 14% for 16:0 RhP, 32% for TF (t), and 51% for 18:1 RhP). The differences in stability of the labeling of LPs, likely result from the degree of saturation of the fluorescent phospholipids. Despite the shorter acyl chain length, 16:0 RhP label is more stably associated with LP compared to 18:1 RhP. Clearly, the length of lipid chain of the labeled phospholipid is less important for labeling stability in LPs compared to being saturated or not. Moreover, the position of fluorophore attachment to the phospholipid affects dissociation; tail‐labeled TF (t) showed a significantly higher dissociation percentage compared to head‐labeled TF (h) (On average: 32% vs 7%), consisting with previous observation.^[^ ^40^ ^]^ However, for the same unsaturated 18:1 PE lipid coupled to different fluorophores, the detachment of TF (h) was substantially lower than that of 18:1 RhP (On average: 7% vs 51%) (Figure 3b). The high dissociation of rhodamine PE may be due to the light‐induced propensity of rhodamine to promote peroxide production and lipid oxidation, and thus formation of membrane domains.^[^ ^41^ ^]^ These dissociation values are in good agreement with previous findings,^[^ ^8^, ^42^, ^43^ ^]^ and indicate that the inherent properties of the label may have an effect on the stability of LP labeling, as well as the accuracy of tracking NPs.

To verify that, detachment of fluorescent lipids is not attributable to the double‐labeling approach, we compared the dissociation of the most instable label, 18:1 RhP, from SFLPs and DFLPs incubated in serum and HEPES (Figure S5a, Supporting Information). We observed no significant difference between the two formulations with respect to dissociation under the same conditions. Notably, we found that labeling stability of DFLPs in serum is both time‐dependent (1 h vs 24 h: *p* < 0.001) (Figure S5c,d, Supporting Information) and serum concentration‐dependent (10% vs 100% serum: *p* < 0.001) (Figure S5e,f, Supporting Information). This indicates that several possibilities could be considered to enhance the reliability of fluorescent‐based observations. In vitro, serum concentration could be decreased to prevent the removal of labels from LPs by serum components, while also incubation time can be shortened. However, to enable full‐scale spatiotemporal delineation of NPs both in vitro and in vivo, we advocate, by choosing the appropriate label and by changing labeling strategy, dual labeling of NPs. Additionally, to corroborate the dissociation results from the intensity‐based area evaluation, the label dissociation was calculated based on the average label‐to‐lipid ratio of FLPs (3–4.5 mL of elution volume, see method 4.6) (Equation (2)) (Figure 3b, negative y‐axis). Interestingly, the two dissociation evaluation methods showed such good agreement, suggesting that the majority of FLPs remained intact and were successfully eluted in the first peak. To summarize, the dual‐labeling strategy does not change the labeling stability, besides extrinsic conditions, intrinsic properties of the lipid marker contributed to the labeling instability, including covalently linked location of fluorophores to the chosen lipid, the selection of fluorophores that connect to lipid, and the lipid saturation extend.

### 2.4 Mass of Components in FLPs Reveals that the Majority of NP Remain Intact and Do Not Contribute to the Leaking of Labels

**Figure 4:**
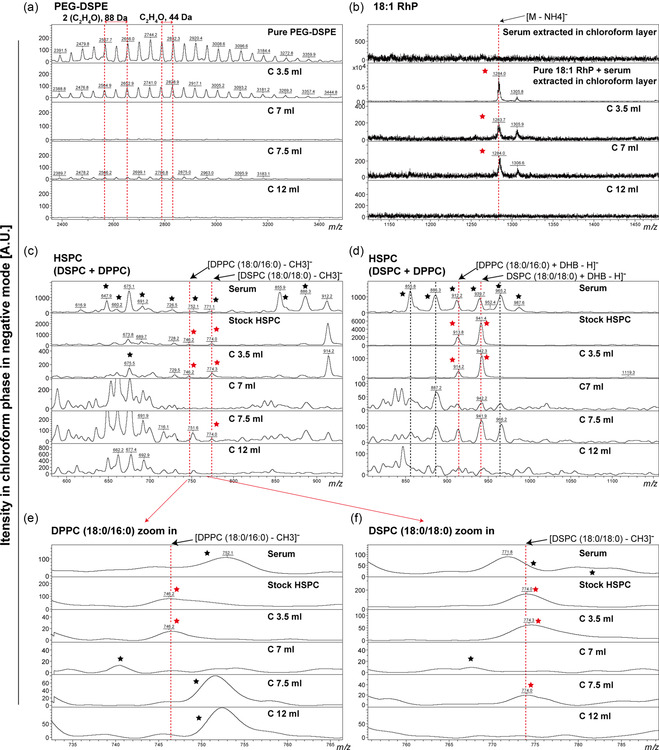
Negative‐ion mass spectra of an extracted lipid mixture (eluent of 18:1 RhP‐L in serum) in chloroform phase. a) Mass spectra of PEG‐DSPE, repeating units separated by 44 Da (the adjacent two clusters are denoted by two red dashed lines), resulting from the loss of C_2_H_4_O (ethylene oxide) blocks. b) The mass of the 18:1 RhP [M − NH_4_]^−^ ion was determined to be around *m/z* 1284 (isotope averaged values), using a pure molecule as a reference. c) Mass of HSPC (a mixture of 11.4% DPPC and 88.6% DSPC), DSPC 18:0/18:0 [M − CH_3_]^−^ and DPPC 18:0/16:0 [M − CH_3_]^−^ mass spectra, using stock HSPC and serum as positive and noise control, respectively. d) The fragment ions at *m/z* 942 and 914 were classified as [DSPC18:0/18:0 + DHB − H]^−^ and [DPPC18:0/16:0 + DHB − H]^−^. This was accomplished by adding DHB to the HSPC molecule, followed by dehydrogenation. e) Magnification of the mass spectrum of the DPPC 18:0/16:0 [M − CH_3_]^−^ ion at *m/z* 746. f) Zoom in on the mass spectrum of the DSPC 18:0/18:0 [M − CH_3_]^−^ ion at *m/z* = 774; C3.5, C7, C7.5, and C12 represent the chloroform layer extracted samples in corresponding elution volume. The red dashed lines indicate the area of interest. The red stars indicate the presence of a positive signal at the red dashed line, whereas the black stars and dashed lines indicate the presence of serum noise.

We hypothesize that the integrity of LP remains intact when the dissociation of lipid markers happens. If LPs do degrade, we expect to observe substantial amounts of FLP components (e.g., HSPC, PEG‐DSPE) along with detached lipid markers in the second SEC peak (elution volume of 7 mL). To test this hypothesis, we used MALDI‐TOF‐MS to determine the mass of the molecules that comprise FLP per elution fraction. All eluted samples were extracted in chloroform/methanol/water phase using Bligh and Dyer's method.^[^ ^23^ ^]^ By comparing fluorescence from the lipid markers under UV light (Figure S6, Supporting Information), it is obvious that the fluorescent lipids were present in the chloroform phase and were absent in the water phase. The lipid extraction was validated further by examining the mass of interest in the chloroform and water phases. First, to determine the structural stability of pure fluorescent lipids after 24 h of incubation in 100% serum at 37 °C, the two most detachable fluorescent markers 18:1 RhP and TF (t) were detected in negative ion mode. The fractions around *m/z* 866 (Figure S7a, Supporting Information, red dashed line) and 1,284 (Figure S7b, Supporting Information, red dashed line, isotope averaged values) were identified as TF (t) and 18:1 RhP (Table S1, Supporting Information), respectively, which were formed through the loss of one hydrogen [M–H]^−^ and one ammonium [M–NH_4_]^−^, respectively. When comparing to the pure sample (Figure S7a,b, Supporting Information, second track), the mass of TF (t) and 18:1 RhP appeared quite stable when incubated with serum (Figure S7a,b, Supporting Information, third track), indicating that the labeling instability of LP was not due to structural disintegration of the fluorescent markers. We next used the most exchangeable FLP, 18:1 RhP‐L, as a model to confirm the lipid marker exchange in the presence of serum. As expected, the mass of 18:1 RhP extracted in chloroform was constant in retention volumes 3 to 10 mL (0.5 mL per fraction) (**Figure** **4**b, S8a, Supporting Information), confirming the presence of 18:1 RhP as observed by SEC (Figure S8b, Supporting Information).

Additionally, in positive mode, fragment ions in serum with masses (Figure 2a, dark stars) coexist with masses in stock HSPC (supplied by manufacturer as a mixture of 11.4% DPPC and 88.6% DSPC),^[^ ^44^ ^]^ which makes it difficult to distinguish liposomal HSPC from lipids in serum. Therefore, within the same 18:1 RhP‐L model, we examined mass distribution of HSPC and PEG‐DSPE in the chloroform phase with negative mode. The negative fragment ions at *m/z* 774 and 746 (Table S1, Supporting Information, Figure 4c, Figure S9a, Supporting Information, red dashed lines) corresponded to the ions [DSPC 18:0/18:0 – CH_3_]^−^ and [DPPC 18:0/16:0 – CH_3_]^−^, respectively. These two species result from the loss of a methyl group from the phosphate head group.^[^ ^45^, ^46^ ^]^ When compared to serum mass peaks, the high signal at *m/z* 774 (Figure 4f, S10a, Supporting Information, red dashed line) appeared only in elution volumes 3–5.5 mL, and relatively little ion intensity was detected at 7.5 mL. However, due to the overlap of serum masses in volumes 5–12 mL, the signal at *m/z* 746 (Figure 4e, S10b, Supporting Information, red dashed line) can only be identified as DPPC in 3–4.5 mL. A similar result was found when observing the fragment masses obtained at *m/z* 942 and 914 (Table S1, Supporting Information, Figure 4d, S9b, Supporting Information, red dashed lines), which were determined to be [DSPC 18:0/18:0 + DHB–H]^−^ and [DPPC 18:0/16:0 + DHB–H]^−^, respectively, by adding DHB (matrix) to the HSPC molecule followed by one hydrogen loss. In short, HSPC was mostly present in volumes 3–5.5 mL according to the elution profile, with just a single peak of DSPC 18:0/18:0 detected at 7.5 mL.

The masses of PEG‐DSPE were observed as a repeat of peaks with masses separated by 44 Da (Figure 4a, S11a, Supporting Information, red dashed line), which can be explained by the mass of C_2_H_4_O (Ethylene oxide) monomer building blocks of PEG.^[^ ^47^ ^]^ These repetitive peaks with an offset of 44 Da were observed in 3 to 5.5 mL and also in 7.5 mL, which was consistent with the distribution of HSPC. Additionally, 18:1 RhP, HSPC, and PEG‐DSPE were absent in the water phase (Figure S11b, S12, Supporting Information), showing that the lipids in each sample were extracted by chloroform optimally, enabling the accurate determination of lipids distribution. In conclusion, comparing the broad mass distribution of 18:1 RhP (0.1% of total lipid), when FLP was incubated in serum and eluted by SEC, the majority of HSPC and PEG‐DSPE (60% of total lipid) were present in the first peak of SEC profile, indicating that the primary structure of LP was not compromised under serum conditions. A low signal of HSPC and PEG‐DSPE was detected in an elution volume of 7.5 mL, which may be marginally released from FLP and eluted in the second peak because of interaction with proteins in serum, such as BSA, ApoA1, ApoE.^[^ ^28^, ^48^ ^]^ Together we conclude that label detachment from FLPs is most likely due to high affinity to serum compounds like transport proteins, instead from the disintegration of LPs.

### 2.5 Dual‐Labeling Strategy Demonstrated that Dissociation of Fluorescent Label from LPs Misdirects Intracellular Localization

As described above, the dissociation of fluorescent markers from FLPs occurred mostly in serum. Since the intracellular environment might have a comparable effect, we argue that use of single fluorescently labeled LPs is insufficient for live cell imaging; detection of fluorescence coming from a pixel in a cell needs to be a univocal indicator of an actual NP in that pixel. Therefore, we employed a dual labeling strategy with the rational that when both labels are present in one pixel over time this is a strong indication that the intact NP is there. To underscore this argument, we used TF (t) (TopFluor PE tail conjugated)/DiD double‐labeled LPs as dual‐labeling example (DFLP). These two fluorophores have significantly different stability and spectra, which permit analysis by flow cytometry and fluorescence microscopy.

#### 2.5.1 Intensity‐Based Analysis by Flow Cytometry is Insufficient for Quantifying Cellular Uptake of FLPs

**Figure 5:**
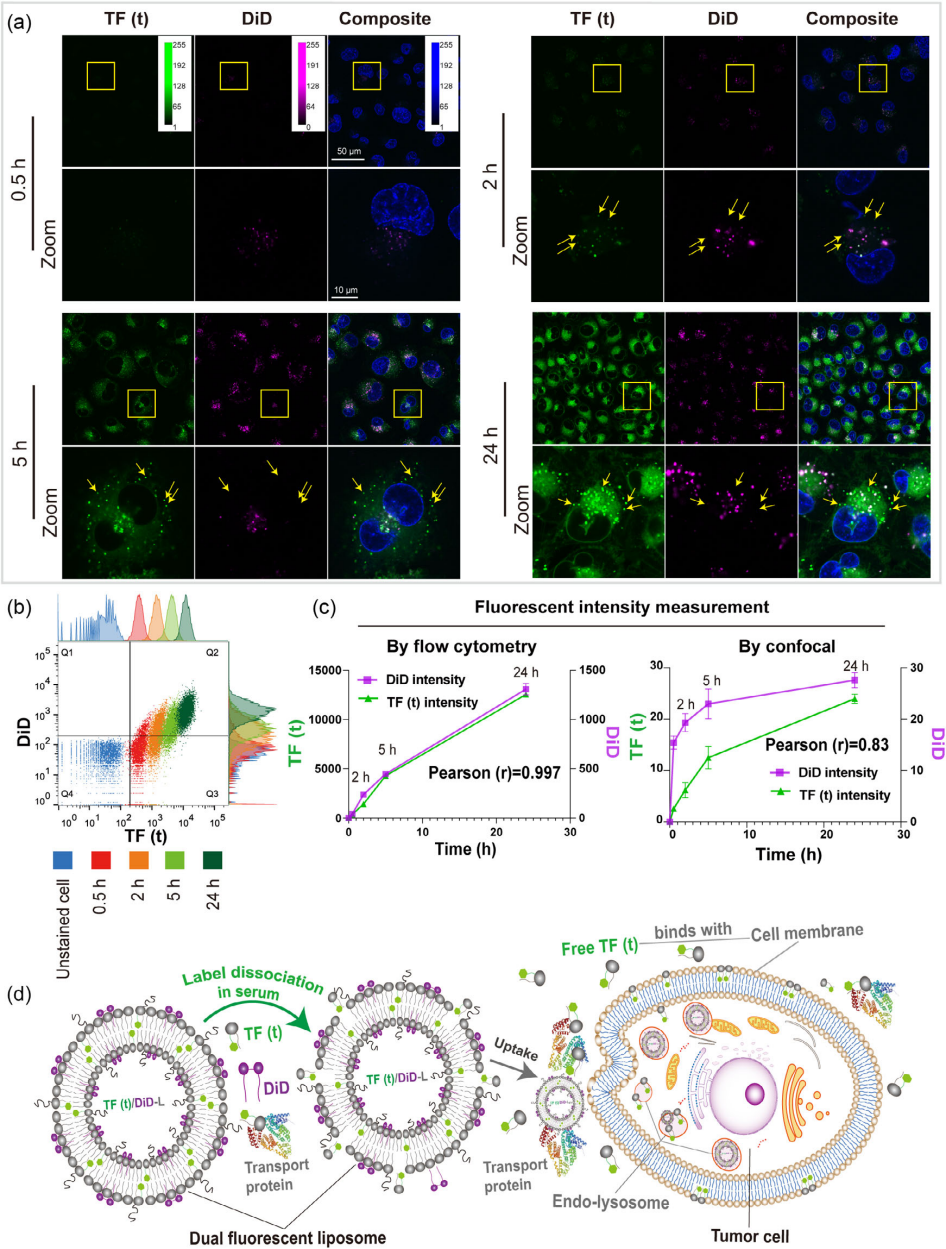
Quantification of dual FLPs [TF(t)/DiD‐L)] uptake by human melanoma BRO lung metastasis (BLM). a) Fluorescent confocal images acquired throughout time. The yellow rectangles depict the cells in the magnified image, and the yellow arrows indicate the absence of colocalization between TF (t) and DiD channels. Setting: 63 × NA1.4 oil immersion objective lens, TF (t) channel: laser 488, 1%; photomultipliers tubes (PMT) gain, 650; DiD channel: laser 633, 4%; Hybrid Detector (HYD) gain, 300; Hoechst channel: laser 405, 1%; PMT gain, 800. b) Time‐combined uptake plot of flow cytometry by FlowJo software, populations were gated based on the positive fluorescence intensity of TF (t) and DiD. c) Mean fluorescence intensity of two labels populations at different time points measured by flow cytometry (left) and fluorescent confocal (right). Pearson correlation coefficients (r) were used to determine the correlation between two label signals. Data represent mean ± S.E.M. of three images from three separate experiments. d) Schematic illustration of DFLPs uptake process by a tumor cell.

First, we determined the time‐dependent uptake of DFLPs by flow cytometry. We expect distinct cellular uptake profiles of the two fluorescent markers considering the difference in labeling stability. Human melanoma BRO lung metastasis (BLM) cells were incubated with DFLPs at a final concentration of 1.2 mm in complete culture media containing 10% bovine serum (Figure S1d, Supporting Information), and harvested at different time points of 0.5, 2, 5, and 24 h. Over time an increase in both fluorescence signals is observed by flow cytometry (**Figure** **5**b,c, S13a, Supporting Information), indicating an increase in DFLP uptake by cells and a high degree of coherence (Pearson correlation coefficients: 0.997) between the two labels. These results seem to indicate a good stability of labeling of NPs during interaction with cells. However, similar results were obtained when cells were incubated with premixed equal amounts of single FLPs labeled with TF (t) or DiD, at a final concentration of 1.2 mm (Figure S13b,d, Supporting Information). Therefore, we argue that the intensity‐based observation by flow cytometry can only tell an overall tendency of the fluorescent signal, but does not provide data on whether the labels are still associated with the NP. It is important to be able to differentiate between two separated signals coming from different locations within a cell and coexisting signals coming from the same location when defining the intracellular uptake process.

**Figure 6:**
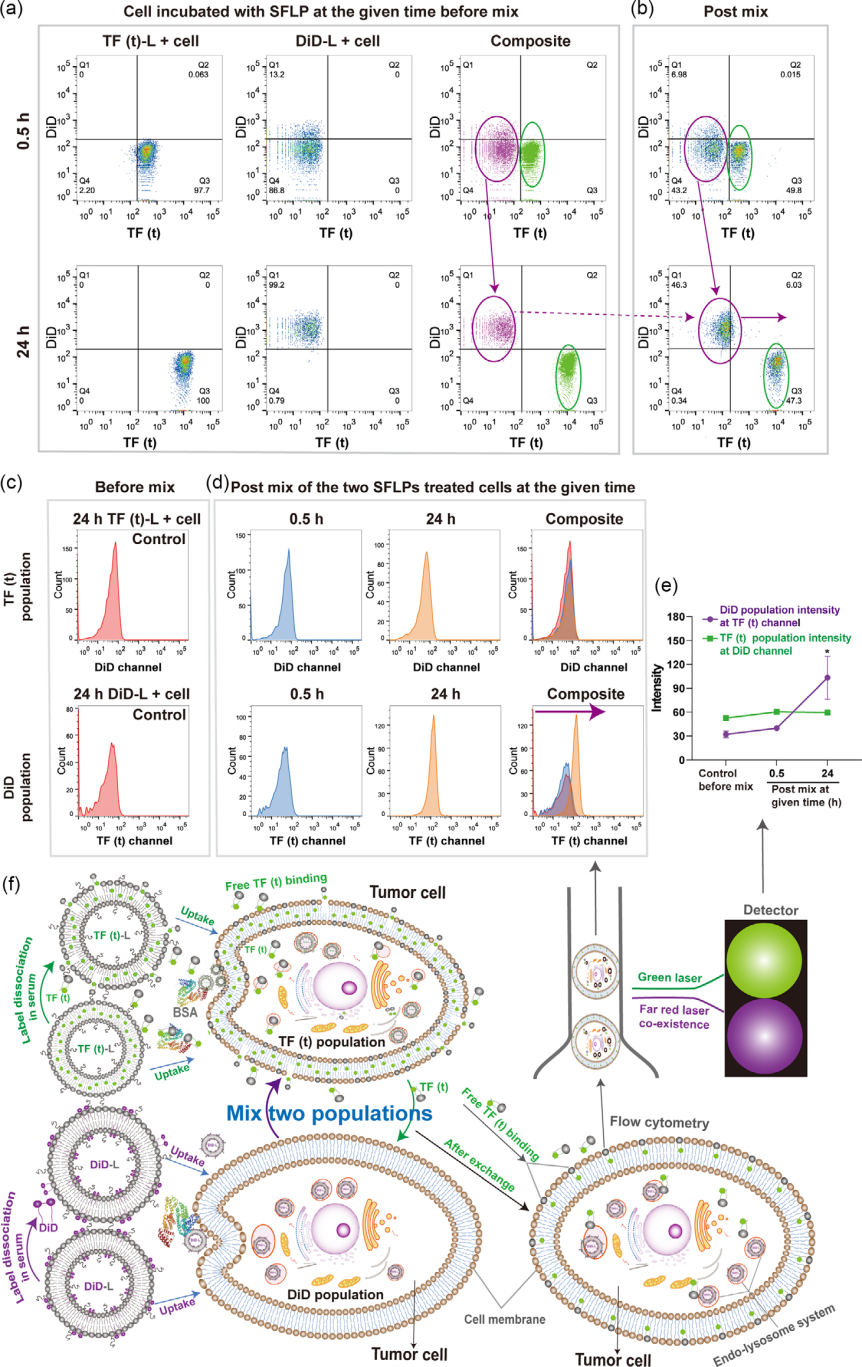
Flow cytometry analysis of post‐mixed cells incubated with singly labelled FLPs (SFLPs) [TF (t)‐L or DiD‐L]. a) Equal amount of two type of SFLPs were separately taken up by cells for 0.5 and 24 h and detected. The predicted distribution of these two sub‐populations was plotted in composite image by FlowJo. The purple and green circle denote DiD and TF (t) populations, respectively. b) The two SFLPs‐treated cell populations at the same time were washed and mixed for 30 mins, followed by flow cytometry. The DiD population showed a right shift toward the TF (t) population. c) Histogram of each SFLP treated population before mixing, with 24 h SFLP‐incubated cells serving as a control. d) Histogram of the two SFLPs treated populations (exposed for 0.5 or 24 h) shows increased signal in the TF (t) channel of the DiD population. e) Intensity measurement based on flow cytometry results. Kruskal–Wallis with multiple comparisons was used to analyze these data. The error bars represent the S.E.M., *n* = 3, *p < 0.05, for comparisons with control within each subgroup. f) Pictorial image of post‐mix experiment of two SFLPs treated cells.

We further tested whether detached labels could cross‐contaminate cells which are not prior exposed to these FLPs. Cells were treated with an equal amount (final concentration of 1.2 mm) of single TF (t)‐L or single DiD‐L separately for 30 min and 24 h and washed with PBS. Then, the two different single FLPs‐treated cell populations were mixed for 30 min followed by flow cytometry (**Figure** **6**f). If there was no transfer of label, an identical distribution of these two cell populations [with TF (t) or DiD] before (Figure 6a, composite) and after the mix should be obtained (Figure 6b, post mix). However, the DiD‐L‐incubated cell population (purple circle) right‐shifted to the TF (t) population (Figure 6b, post mix, green circle) when compared to unmixed setting (Figure 6a, composite), which was especially noticeable at 24 h of incubation (Figure 6c,e). This result indicates that TF (t) transferred to cells of the DiD‐L‐treated population. Apparently, transfer of DiD was marginal as we did not detect by flow cytometry measurable increase of DiD signal in TF (t) population (Figure 6c,e). This result confirmed the relative labeling stability of DiD in SFLPs, also, when taken up by cells. In conclusion, fluorescent label separation from LPs occurs during cellular processing, and is then followed by association with lipid‐rich compartments. More so, our results indicate that intensity‐based observation by flow cytometry is not sensitive enough to demonstrate labeling stability and to evaluate the uptake of NPs. Consequently, fluorescence microscopy was utilized to further clarify the impact of labeling stability on NPs tracking.

#### 2.5.2 Intracellular Observation of DFLPs by Confocal Microscopy Shows that Label Dissociation Causes False Positive Location Determination

Incubating BLM cells with DFLPs [TF (t)/DiD‐L] is followed by confocal fluorescence microscopy demonstrated comparable fluorescent intensity results (Pearson correlation coefficients: 0.83) compared to flow cytometry (Figure 5c); both labels appeared to be slightly detectable after only 0.5 h of incubation, followed by continuous increase in brightness of fluorescence. Nevertheless, confocal microscopy imaging showed pixels of TF (t) and DiD that did not colocalize (Figure 5a, yellow arrow). Already at an early time point, we observed the separation of the green and purple channels indicating the dissociation of the fluorescent labels from the LP. Green fluorescence showed abundant colocalization with cellular structures, which may be a result of dissociation of the lipid markers in the extracellular environment followed by independent uptake, or separation from LPs after uptake in the cell. These results suggest that due to the false positive signal from detached labels, the single molecular tracking results by high‐resolution confocal microscopy does not correlate with the overall intensity‐based measurements.

Together our results confirm that due to the intricate intracellular environment, labeling stability quantification and free label purification ex vivo could not guarantee the accuracy uptake process; for intracellular studies using fluorescence microscopy, single labeling of LPs likely provides false information on localization of the NP in the cell. Therefore, pixel‐based colocalization analysis of two labels on DFLPs was further performed in live cells by high‐resolution confocal microscopy.

### 2.6 Dual‐Labelling Strategy is a Prerequisite for Precise Localization of Intracellular NPs by High‐Resolution Microscopy

#### 2.6.1 Colocalization Analysis of DFLPs Correlated Well with Ex vivo Labeling Stability Results

**Figure 7:**
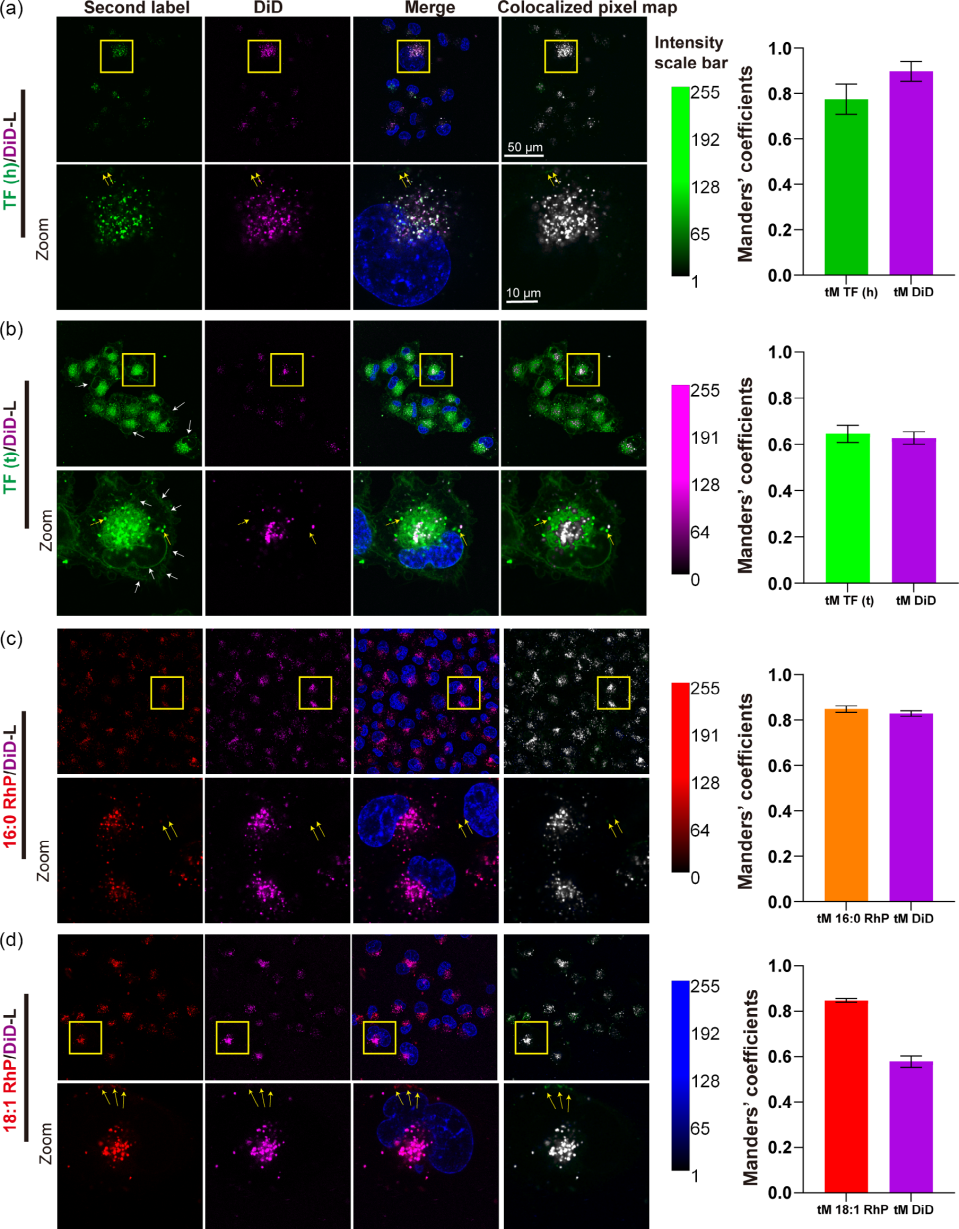
Colocalization analysis of the two labels in DFLPs after uptake by BLM for 24 h. a–d) Fluorescence image of each DFLP. The yellow rectangles depict the cell in the zoomed‐in image, and the yellow arrows indicate the intensity that is not co‐localized with the DiD channel. For the co‐localized pixel map, DiD is defined as the blue color for all groups, while the second label [TF (h), TF (t), 16:0 RhP and 18:1 RhP] are defined as green. The pixel with white color in the pixel map symbolizes co‐localized intensity. Confocal setting: 63 × NA1.4 oil immersion objective lens; TF (t) channel: laser 488; 1%; PMT gain, 650; TF (h) channel: laser 488, 2%, PMT gain, 800. 18:1 RhP channel: laser 561, 0.5%; HYD gain: 50. 16:0 RhP channel: laser 561, 0.5%; HYD gain, 200. DiD channel: laser 633, 8%; HYD gain 300; Hoechst laser channel: 405, 1%; PMT gain 800. e–h) Histogram of Manders' colocalization coefficients relative to DFLPs in the left panel. Complete data are reported in Table S3, Supporting Information. Data represent mean ± S.E.M. of at least 15 cells of 3 individual experiments.

As intracellularly dissociated labels accumulate at distinct localizations depending on the nature of the label, we proposed that for the two labels on DFLPs, cellular colocalization observed by fluorescent confocal microscopy is proportional to the labeling stability evaluated by SEC result. Therefore, we cultured BLM cells with different DFLPs for 24 h and quantified colocalization of the two fluorescent labels. Manders’ colocalization coefficients (MCC) and Pearson's correlation coefficient (PCC) were used for colocalization analysis of each DFLP group. Indeed, PCC of TF (h)/DiD‐L, 16:0 RhP/DiD‐L, 18:1 RhP/DiD‐L, and TF (t)/DiD‐L, declined from 0.82 to 0.69, and a similar distribution of Manders’ coefficient was observed (**Figure** **7**, Table S3, Supporting Information, Manders’ tM 1 and tM 2). It is important to note that, despite the high‐labeling stability of DiD, this label exhibits lower photostability compared to TopFluor and rhodamine. We observed declining fluorescence intensity during time‐lapse imaging with short intervals **(**Figure S14, Supporting Information, white arrow, Supplementary Movie 1, Supporting Information). This could impact the detection of NP localization and long‐term single particle tracking in live cells when exploring single‐dye‐labeled NPs (see supplemental discussion 2). Moreover, despite the good labeling stability of NBD PE, the fluorescent intensity to noise ratio of this label is too low for optimal live cell imaging by fluorescent microscopy, preventing us from evaluating colocalization in the NBD PE/DiD‐L group. Strikingly, when combining the fluorescence image and the colocalization result, 18:1 RhP (Figure 7d,h) showed a better correlation with DiD than TF (t) (Figure 7b,f), which should be the opposite given the better ex vivo labeling stability result of TF (t) than 18:1 RhP (Figure 3b). The poor colocalization of TF (t) with DiD is likely explained by the relatively strong interaction of TF (t) with cellular and organelle membranes (Figure 7b, white arrows), which moved with a fluid‐like motion together with the attached free TF (t) (Figure S14, Supporting Information, yellow arrow, Supplementary Movie 1, Supporting Information). This result suggests that the interaction of fluorescent labels with cellular structures and compounds affects labeling stability and determination of NP localization. While the good colocalization for 18:1 RhP with DiD (Figure 7d, Merge) might attribute to the shared uptake mechanism of the highly detached free dye (18:1 RhP) with 18:1 RhP/DiD‐L, and longer incubation (24 h) may therefore result in accumulation at the same location, such as the endolysosomal system.

This was further demonstrated by 24 h live cell confocal microscopy of the 18: 1 RhP/DiD‐L in BLM cells where lysosomes were prelabeled with CellLight Lysosomes‐GFP (BacMam 2.0). As demonstrated by the results, only a few cells exhibited a coherent signal distribution (Figure S15, Supporting Information, yellow arrows) between the 18:1 RhP and DiD channels over time, while majority of cells exhibited a quicker and more visible 18:1 RhP signal (mostly located in lysosomes). A similar outcome was observed in HS578T (breast cancer cell line) when treated with 18:1 RhP/DiD‐L for 1.5 h (Figure S16, Supporting Information). Overall, these nonaligned correlation results between two labels further confirm our conclusion of co‐uptake pathways of both free labels and NPs, and are also in agreement with previous reports.^[^ ^18^, ^49^ ^]^ Furthermore, it is difficult to determine whether individual labels are separated from NPs by motion characteristics of the signal, highlighting the complexity of determining nanoparticle localization and NP tracking in cells by individual label, as well as the necessity of dual‐labeling strategy.

#### 2.6.2 Dual‐Labeling Strategy is Beneficial for Precise Localization and Tracking of LPs at Single Molecule Level

**Figure 8:**
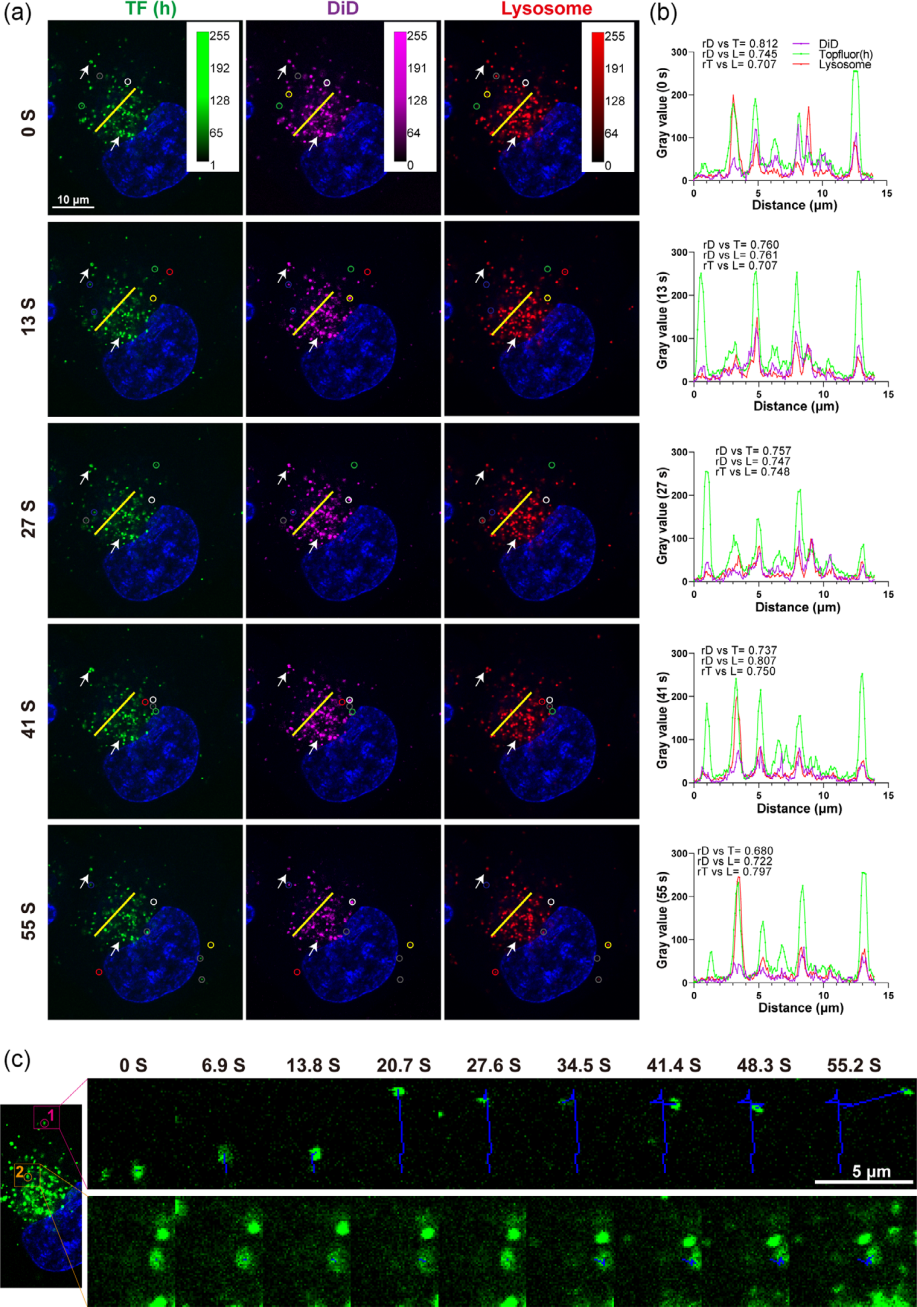
Intracellular correlation analysis between two labels in DFLPs and lysosomes in BLM tumor cells. a) TF (h)/DiD‐L was incubated with cells for 24 h, washed with PBS, fluorescent microscopy was then used to create a short time interval movie. The figure depicts representative images at 0, 13, 27, 41, and 55 s. The white arrows indicate representative examples of both dyes localization in lysosomes. The yellow small circle indicates colocalization of DiD with lysosomes but not with TF (h). The blue small circle indicates colocalization of DiD with TF (h) but not with lysosomes; and gray small circle indicates colocalization of TF (h) with lysosomes but not with DiD. The little circles in green, white, and red depict the signal placement alone of TF (h), DiD, and lysosome marker, respectively. b) Fiji was used for quantitative correlation analysis, and the plot profile corresponds to the yellow lines in the fluorescence images at each time point. The Spearman correlation (r) was used to determine the relationship between dyes. c) Representative single particle tracking over time using the TopFluor channel as an example. The images in the upper right pannel and lower right pannel represent the red and orange rectangles in the zoomed‐in area in the left image, respectively, as well as the NP tracking from the red circle and orange circle. The tracking route is indicated with a blue line. The movie can be found in Movie S3, Supporting Information. Abbreviation: D, DiD; T, TF (h); L, lysosome. Confocal setting: 63 × NA1.4 oil immersion objective lens, TF (h) channel: laser 488, 2%; PMT gain, 800; DiD channel: laser 633, 8%; HYD gain, 300; Hoechst channel: laser 405, 1%; PMT gain, 800; Lysosome channel: laser 561, 1%; HYD gain, 150. Interval time: 6.9 s, total time: 55 s.

When we evaluated the intracellular colocalization of two labels on DFLPs, we note that even for the DFLPs with the highest labeling stability, colocalization did not reach 100%, and noncolocalized signals were seen (Figure 7a–d, yellow arrows). Correlation analysis represents not only pixel‐based overlap but also proportional distribution within cellular components of the two probes, which, with a good correlation over time, indicates that the two probes are located in the same compartment.^[^ ^50^ ^]^ Therefore, we tested the reliability of DFLPs by using correlation analysis under fluorescent microscopy at the live‐time molecule level. We selected the most stable formulation, TF (h) (TopFluor PE head group conjugated)/DiD‐L, as a test system for observing how well DFLPs interacted with lysosomes that were stained with LysoView 540. Furthermore, we utilized the Spearman rank (r) correlation to quantify colocalization of these two dyes on DFLPs with lysosomes. Intracellular trafficking of TF (h)/DiD‐L in BLM tumor cells after 24 h demonstrates a high Spearman correlation of around 0.7 among TF (h), DiD, and lysosome signals, even over short‐time intervals (**Figure** **8**, Supplemental Movie 2, Supporting Information). The good alignment suggests that the majority of these DOXIL‐like DFLPs in lysosomes remained intact even after 24 h incubation, which might be the primary reason for the poor efficacy of DOXIL,^[^ ^51^ ^]^ highlighting the relevancy of double labeling for single NP tracking and the need to unravel unsatisfactory delivery. Interestingly, by performing single LP tracking, we found that LP located in the peripheral area (ROI 1, red circle) showed more dynamic movement, while the LP adjacent to the nucleus ((ROI 2, orange circle) showed less movement (Figure 8c, Supplementary Movie 3, Supporting Information), consistent with the previous result.^[^ ^52^, ^53^ ^]^ Moreover, as illustrated in Figure 8a, the white arrow indicates complete colocalization of TF (h), DiD, and lysosomes over time, indicating the good combination of these two labels in LPs. However, there was also evidence that TF (h), DiD, and lysosome signals did not colocalize, or only two colocalized (Figure 8a, circles with different colors). This may be due to DFLPs being processed in the cell followed by label separation and followed by accumulation in distinct lysosomes or other tumor cellular compartments. This attribute, the label separation time from DFLPs, might be utilized as an estimation of the integrity of the LP and to visualize when and where degradation may occur, as well as an indication of payload release. In conclusion, this result emphasizes the critical need for the application of double‐labeled NPs, which might bolster the authenticity in terms of objective detection of localization, particularly for super‐resolution live cell imaging, of NP uptake and processing.

## 3 Conclusion

Nanoparticles, and in particular lipid‐based NPs such as liposomes (LPs) and lipid nanoparticles (LNPs), have been the subject of substantial research as potential carriers of active compounds for therapy, diagnosis, and combination thereof.^[^ ^1^, ^2^, ^3^ ^]^ While the formulation of compounds in NPs provides many advantages for application in patient‐tailored treatment, the working mechanism, and especially the fate of NP and compound, e.g., a chemotherapeutic, becomes more complicated. A crucial element in compound delivery for precision medicine by NPs is the eventual destination of the NP in a cell and the delivery of content at the right site. Proper evaluation of the intracellular fate of carriers is imperative for clinical translation. The clinical relevance of precise determination of NP fate, and thus our dual‐labeling approach, is underscored by the recently developed mRNA vaccines (BioNTech‐Pfizer and Moderna);^[^ ^54^ ^]^ how these lipid NPs are taken up by cells and how NPs behave in endosomes, to allow mRNA to escape to the cytosolic compartment, determine the treatment outcome.^[^ ^55^ ^]^

Our findings underscore the potential risks associated with insufficient testing of the integrity of nanocarriers and the stability of labeling. Straightforward intensity‐based tests do not suffice. Here, we show and prove the importance of dual labeling strategy in the observation of NPs at the subcellular level. Our work, we believe, may give insight into procedures for designing other lipid‐based platforms for fluorescence‐guided targeted delivery and will assist in determining whether drugs loaded in LP reach the expected place of action. Due to the dynamic nature of cells, we advocate not only screening for labels with a lower affinity for the biological environment but also utilizing two distinct types of lipid labels, particularly when carriers lack control to indicate integrity. In contrast, when relatively stable double labeling is employed for long‐term live cell imaging, highly colocalized signals overtime on a carrier indicate intact uptake. Alternatively, occurrence and level of noncolocalization (i.e., dissociation) over time may be used to evaluate NP degradation and drug release from NPs. Finally, the dissociation of the two fluorescent labels may give insight into the separate destiny of components of disintegrated vesicles. Taken together, our findings not only provide lessons useful for confocal (super‐resolution) imaging of intracellular trafficking of NPs but may also provide insight in nanodrug platform processing for enhanced precision medicine.

## 4 Experimental Section

4.1

4.1.1

##### Materials

1,2‐distearoyl‐sn‐glycero‐3‐phosphoethanolamine‐*N*‐[methoxy(polyethylene glycol)‐2000] (DSPE‐ PEG2000), l‐α‐phosphatidylcholine, hydrogenated (Soy) [Hydro Soy PC, HSPC, a mixture of 11.4% 1,2‐dipalmitoyl‐sn‐glycero‐3‐phosphocholine (DPPC) and 88.6% 1,2‐distearoyl‐sn‐glycero‐3‐phosphocholine (DSPC)], 1,2‐dioleoyl‐sn‐glycero‐3‐phospho‐ethanolamine‐N [(dipyrrometheneboron difluoride) butanoyl] [18:1 TopFluorPE, TF (h)], 1‐palmitoyl‐2‐(dipyrrometheneboron difluoride) undecanoyl‐sn‐glycero‐3‐phosphoethanolamine [TopFluor PE, TF (t)], 1,2‐distearoyl‐sn‐glycero‐3‐phosphoethanolamine‐*N*‐ (7‐nitro‐2‐1,3‐benzoxadiazol‐4‐yl) [18:0 NBD PE (NBD‐DSPE)], 1,2‐dioleoyl‐sn‐glycero‐3‐phosphoethanolamine‐*N*‐(lissamine rhodamine B sulfonyl) (ammonium salt) (18:1 Liss Rhod PE, 18:1 RhP), 1,2‐dipalmitoyl‐sn‐glycero‐3‐phosphoethanolamine‐*N*‐(lissamine rhodamine B sulfonyl) (ammonium salt) (16:0 Liss Rhod PE, 16:0 RhP), and cholesterol were purchased from Avanti Polar Lipids. Vybrant DiD, Micro BCA Protein Assay Kit, and CellLight Lysosomes‐GFP (BacMam 2.0) were purchased from Thermo Fisher Scientific. Sigma Aldrich provided HEPES, MTT, fetal bovine serum (FBS), NaCl, and DMSO. Fisher Scientific supplied CL‐4B Sepharose. The cell culture medium used in all procedures described in this article is DMEM without phenol red (PAN‐Biotech, Germany). VWR provided LysoView 540 and chloroform. Bruker Daltonics provided the matrix CHCA (alpha‐cyano‐4‐hydroxycinnamic acid) and (2,5‐dihydroxybenzoic acid, DHB).

##### Fluorescently Liposome (FLP) Preparation

Plain LPs were produced using previously published methods.^[^ ^56^ ^]^ NPs were made of HSPC: DSPE‐PEG2000: Cholesterol at a molar ratio of 55:5:40. In brief, 50 μmol lipids were dissolved in chloroform and then dried overnight at 40 °C using a rotator–evaporator system. At 60 °C, HEPES buffer (10 mm HEPES, 150 mm NaCl buffer, pH 7.4) was used to rehydrate the dry lipid film. The size was controlled to around 100 nm by first sonicating for 5 min followed by extruding 11 times through Nuclepore membrane (Whatman Inc, USA) with pore sizes of 200, 100, and 50 nm, respectively. All FLPs were made by adding fluorescent labels to the lipids mixture in chloroform at a concentration of 0.1 mol%, except DiD, which is at a concentration of 0.005 mol%. ZetaSizer Nano‐ZS (Malvern Instruments Ltd., UK) was used to determine the LP diameter (size), polydispersity index (PDI), and zeta potential.

##### Cytotoxicity Assay

As previously reported, the toxicity of plain LP was determined using the MTT technique.^[^ ^57^ ^]^ In brief, human BLM melanoma cells were plated in 96‐well plates for 24 h at a density of 6,000 cells per well, followed by the addition of plain LPs at concentrations ranging from 0–4 mm and incubated for another 24 h. Following that, MTT (3 mg mL^−1^, 50 μL) was added to each well for 3 h at 37 °C, the medium was withdrawn, and another DMSO (100 μL) was added to dissolve formazan. At 510 nm, the optical density (O.D.) was determined using a Wallac Victor Plate Reader. Cell viability was determined as a percentage of control cells.

##### Size Exclusion Chromatography

Separation of dissociated labels was accomplished using the approach described in a previous article.^[^ ^8^ ^]^ To summarize, FLPs (300 μL, 2 mm) were mixed with HEPES or serum (10% or 100% depending on the experiment setting) and incubated for 24 h at 37 °C, followed by elution through a column [Cytiva (Pharmacia) C 10/150 mm] loaded with Sepharose CL‐4B and attached to a MINIPULS3 Peristaltic Pump (Gilson) at a flow rate of 750 μL min^−1^ with HEPES buffer. Each eluted fraction (500 μL) was collected in an Eppendorf tube, and the label separation profile was validated using a Wallac Victor Plate Reader (Table S4, Supporting Information).

##### Dissociation of Fluorescent Labels from LP Calculated by Intensity‐Based Area Analysis

The dissociation of each fluorescent label from the carrier was determined by the percentage of the area remaining after 5.5 mL of retention volume (A 5.5–20) compared to the entire area (A 3–20); our procedure is quite similar to Rasmus Münter's.^[^ ^8^ ^]^ As illustrated in Figure 1c, the black dashed line denotes the diacritical point for calculation, and the following formula is used to count dissociation based on area

(1) $$\text{Dissociation }\left(\text{area}\right)\%=\frac{\text{Sum }A^{\left(5.5-20\right)}}{\text{Sum }A^{\left(3-20\right)}}\times 100\%$$

##### Dissociation of Fluorescent Labels from LP Calculated by Label‐to‐Lipid Ratio

To evaluate label dissociation more accurately, the label‐to‐lipid ratio in the eluted fractions was determined. Each original batch of FLPs was used as a standard curve to determine the label concentration of the eluted fraction using spectrofluorimetry (Hitachi F‐4500 Fluorescence Spectrophotometer, Japan) (Table S5, Supporting Information). After 4.5 mL of elution volume, we identified the presence of phosphorus in serum (Figure S2c,d, Supporting Information), which may affect phosphorus‐based determination of phospholipid concentration. Therefore, dissociation was determined as follows: the average label‐to‐lipid ratio (*X* ^3–4.5^) of FLP in elution volumes 3 to 4.5 mL was divided by the original ratio *Y* (0.005 mol% for DiD and 0.1 mol% for all other labels) was used to define the label association percentage, which was then subtracted from 1 to calculate the dissociation percentage. The following formula was used to calculate dissociation based on the lipid ratio

(2) $$\text{Dissociation }\left(\text{lipid ratio}\right)\%=\left(1-\frac{\text{Average }X^{\left(3-4.5\right)}}{Y}\right)\times 100\%$$

##### Phosphorus Assay

The phosphorus concentration was determined based on Bartlett's assay.^[^ ^58^ ^]^ In brief, samples (5 μL for freshly prepared liposome, 100 μL for each eluent) or standard phosphorus solution (20–160 μL) were mixed with water (100 μL) in a glass tube and evaporated for 30 min at 140 °C in a blocker heater. Then perchloric acid (300 μL) was added to each tube for 30 mins at 180 °C to destruct the organic structure. After cooling to room temperature, water (1 mL), hexa‐ammonium molybdate (0.4 mL, 1.25%), and ascorbic acid (10%) were added to the sample mixture, followed by shaking and heating in the water bath at 100 °C for 5 min. Finally, tubes were cooled down to room temperature and the absorbance was measured on a Hitachi U3000 spectrophotometer at 797 nm.

##### Flow Cytometry

Flow cytometry was used to determine the internalization of DFLP [TF (t)/DiD‐L] and SFLP [TF (t)‐L and DiD‐L]. In detail, 1 × 10^5^ BLM cells mL^−1^ were seeded in a culture flask for 24 h at 37 °C and 5% CO_2_, and cells were incubated with DFLPs and premixed equal amount of SFLP (final concentration of 1.2 mm), which were used at this concentration to ensure that fluorescent intensity could be detected by flow cytometry at an early time point. While in the premix group, equal amounts (final concentration of 1.2 mm) of TF (t)‐L and DiD‐L were mixed and added to BLM cells. Cells were trypsinized and harvested after 0.5, 2, 5, and 24 h at a final concentration of 5 × 10^6^ cells mL^−1^ in cold PBS (0.05% sodium azide). For the postmix group, the procedure was slightly different, in brief, 1.2 mm TF (t)‐L and 1.2 mm DiD‐L were cultured with BLM cells, respectively. Cells were then harvested at specified time points of 30 min and 24 h, each population with the same concentration at the same time point was then mixed and allowed to settle in cold PBS (0.05% sodium azide) for 30 min. The unmixed SFLP‐treated population at each incubation time point was diluted twice and was used as a control. Finally, the fluorescence intensity of each of the above‐mentioned cells was determined using a FACS Canto II flow cytometer (BD Biosciences, FACS Diva Software) and analyzed using FlowJo v10 software (FLOWJO, LLC).

##### Live Cell Imaging

Fluorescent confocal microscopy was used to determine the intracellular uptake by BLM cells (SP 8, Leica). For uptake of TF (t)/DiD‐L (1.2 mm) that was compared with flow cytometry, cells were cultured in a culture dish (CELLview Culture dish, Greiner) with similar culture conditions. Prior to imaging by fluorescent microscopy, the medium was refreshed at the indicated time points, and then Hoechst 33 342 (1 μg mL^−1^) was added and incubated with cells for 15 min. Finally, cells were imaged in a chamber capable of maintaining 37 °C and 5% CO_2_ at the indicated time points. All live cell imaging was conducted with a 63 × NA1.4 oil immersion objective lens with airy unit 1 as pinhole. The procedure for colocalization analysis imaging of each DFLP was identical to that described above, only after 24 h, and the DFLP concentration used was 0.45 mm, which allows proper visualization.

##### Imaging‐Based Analysis

All live cell confocal imaging analyses were conducted with Fiji. The intensity‐based measurement in Figure 5 was performed after correction for autofluorescence before adding fluorescent NPs. To correct autofluorescence images were obtained with the same confocal setting before adding fluorescent nanoparticles, and these background images were subtracted from the fluorescence images for the intensity analysis. To avoid inaccurate analysis due to heterogeneous intensity distribution, co‐occurrence‐based proper quantitative tools were applied. For colocalization in Figure 7, besides the Pearson's correlation coefficient (PCC), which mainly relies on a good intensity linearity between the two fluorescent probes, we used pixel‐based Manders’ colocalization coefficients (MCC).^[^ ^50^ ^]^ First, a region of interest was drawn, which was then analyzed by a colocalization threshold plugin. The single particle tracking analysis in Figure 8c was performed by a manual tracking plugin. For time‐correlation analysis of the three fluorescent signals in Figure 8, the intensity change in the plotted area of Fiji was measured and then quantified in GraphPad Prism software by Spearman correlation, which is calculated based on signal presence, independently of signal intensity.

##### Lipid Extraction

The lipid extraction method was described by Bligh and Dyer [proportions: chloroform‐methanol‐water: 1:2:0.8 (v/v/v)].^[^ ^23^ ^]^ In brief, for pure fluorescent lipids TF (t) and 18:1 RhP, 5 μL of each sample in chloroform was incubated for 24 h with 100% serum (95 μL) before extraction. Following, chloroform (125 μL) and methanol (250 μL) were added to the above‐mentioned sample and thoroughly mixed for 1 min, followed by the addition of chloroform (125 μL) and vigorous shaking for 1 min. Finally, distilled water (100 μL) was added to the mixture and flowed by vigorous shaking for 1 min. Each mixture was then centrifuged at 4000 g for 5 min at 4 °C, and the solution was separated into three layers, with the upper layer containing polar molecules, the interphase containing some non‐extractable residues, and the lower layer containing lipids in chloroform. As previously stated, 100 μL of each elution sample of 18:1 RhP‐L in serum was used for lipid extraction as well. MALDI‐TOP MS analysis confirms that after extraction, all fluorescent lipids and serum lipids were recovered in the chloroform layer, whereas BSA was detected in the water phase.

##### BCA Protein Assay

Protein concentrations of BSA were determined using the BCA technique.^[^ ^59^ ^]^ In brief, pure BSA was eluted through SEC at 40 mg mL^−1^, the average concentration in serum, and each BSA eluent (150 μL) was mixed well with BCA working regent (150 μL) in a 96‐well plate, followed by incubation at 37 °C for 2 h. Finally, plates were cooled to room temperature before being read on a Wallac Victor Plate Reader for absorbance at 545 nm. The standard curve with the best‐fit polynomial equation was constructed using pure BSA.

##### MALDI‐TOF MS

MALDI‐TOF spectra were acquired on an Ultraflex III MS (Bruker Daltonics, Bremen, Germany) mass spectrometer operating in the positive and negative linear ion modes, respectively, using CHCA (alpha‐cyano‐4‐hydroxycinnamic acid, 10 mg mL^−1^ in water) and DHB (2,5‐dihydroxybenzoic acid, 10 mg mL^−1^ in water) as matrix.^[^ ^60^ ^]^ Direct application of 1 μL droplets of extracted samples to the sample plate was followed by the addition of matrix solution (1 μL). Crystallization was performed under a stream of moderately warm air. Each mass spectrum averaged ,000 single laser shots, with 100 laser shots per location. To produce the best signal‐to‐noise ratio without signal saturation, the laser was set around 10% above the threshold. Except for the mass of BSA and pure serum, which were detected in positive mode with CHCA, the other mass spectra were obtained in the negative mode with DHB as matrix to reduce background serum signal.

##### ESI‐LC‐MS/MS

The samples (eluted in 3.5, 7, and 7.5 mL) after lipid extraction in the water phase were enzymatically digested with trypsin and subsequently measured with a nano‐LC‐Orbitrap MS/MS mass spectrometry system (Ultimate 3000 HPLC, Thermo Fisher Scientific Germering, Germany; Orbitrap Lumos, Thermo Fisher Scientific, San Jose, CA, USA).^[^ ^61^ ^]^ The results were valued based on the bovine dataset. Specifically, 3 μL digest was loaded onto a C18 trap column (C18 PepMap, 300 μm inner diameter (ID) × 5 mm, 5 μm particle size, 100 Å pore size; Thermo Fisher Scientific, The Netherlands) and desalted for 10 min using a flow rate of 20 μL min^−1^ 0.1% TFA. The trap column was switched online with the analytical column (PepMap C18, 75 μm ID × 250 mm, 2 μm particle, and 100 Å pore size; Dionex, The Netherlands) and peptides were eluted with the following binary (A and B) gradient: 4–38% solvent B in 90 min, whereby solvent A consists of acetonitrile (2%) and formic (0.1%) in water and solvent B consists of acetonitrile (80%) and formic acid (0.08%) in water. The column flow rate was set to 300 nL min^−1^.

A data‐dependent acquisition method was used for MS detection: a high‐resolution survey scan from 375 to 1500 Th. was performed in the Orbitrap (value of target of automatic gain control (AGC) 400 000) and a resolution 120 000 at 400 *m/z*; lock mass was set to 445.12003 u (protonated (Si(CH_3_)_2_O)_6_). Based on this survey scan, the most intense ions were consecutively isolated (AGC target set to 10^4^ ions) and fragmented by collision‐activated dissociation (CAD) applying 35% normalized collision energy in the linear ion trap until a duty cycle time of 3 s was reached (top speed mode). After precursors were selected for MS/MS, they were excluded for further MS/MS spectra for 60 s. Data analysis was performed as described in the study of Güzel et al.^[^ ^61^ ^]^

##### Statistical Analysis

GraphPad Prism version 9 was used to perform statistical analysis. All the statistical data are depicted as mean ± standard error of the mean (S.E.M.) from three individual experiments. Analysis of variation of the characteristics of lipid‐based nanoparticles (L‐NPs) was performed with one‐way analysis of variance (ANOVA) with multiple comparisons. Label dissociation analyses were performed using Two‐way ANOVA with multiple comparisons. The statistical analyses of intensity variation from flow cytometry analysis of postmixed cells were conducted using Kruskal–Wallis with multiple comparisons. The significant difference was considered when *p* < 0.05.

## Conflict of Interest

The authors declare no conflict of interest.

## Supporting information

Supplementary Material

Supplementary Material

## Data Availability

The data that support the findings of this study are available from the corresponding author upon reasonable request.
