# Supplementary material for: Precision Localization of Lipid‐Based Nanoparticles by Dual‐Fluorescent Labeling for Accurate and High‐Resolution Imaging in Living Cells
Source: Small Sci. 2023 Jun 27;3(8):2300084. doi: 10.1002/smsc.202300084 (PMC11936020; doi:10.1002/smsc.202300084)
Supplement: Supplementary file 1 — Supplementary Material [file SMSC-3-2300084-s002.pdf]

Precision Localization of Lipid-Based Nanoparticles by Dual-Fluorescent Labeling for Accurate and High-Resolution Imaging in Living Cells

*Wen-Qiu Huang, Peter C. Burgers, Mohamadreza Amin, Theo M. Luider, Timo L. M. ten Hagen**

Wen-Qiu Huang

Precision Medicine in Oncology (PrMiO), Department of Pathology, and Nanomedicine Innovation Center Erasmus (NICE), Erasmus MC Cancer Institute, 3015 GD Rotterdam, the Netherlands.

Peter C. Burgers

Clinical and Cancer Proteomics, Department of Neurology, Erasmus MC, 3015 GD Rotterdam, the Netherlands.

Mohamadreza Amin

Precision Medicine in Oncology (PrMiO), Department of Pathology, and Nanomedicine Innovation Center Erasmus (NICE), Erasmus MC Cancer Institute, 3015 GD Rotterdam, the Netherlands.

Theo M. Luider

Clinical and Cancer Proteomics, Department of Neurology, Erasmus MC, 3015 GD Rotterdam, the Netherlands.

Timo L.M. ten Hagen*

Precision Medicine in Oncology (PrMiO), Department of Pathology, and Nanomedicine Innovation Center Erasmus (NICE), Erasmus MC Cancer Institute, 3015 GD Rotterdam, the Netherlands. Email: t.l.m.tenhagen@erasmusmc.nl. Tel: +31 (0)10 70 43682

Supplementary

Table S1. Proteomic result by ESI-LC-MS in bovine database.

Protein	Accession number	Molecular weight in database	Peptide number (W 3.5 mL)	Peptide number (W 7 mL)	Peptide number (W 7.5 mL)
**Albumin**	P02769	69 kDa	5	79	117
**AFP**	Q3SZ57	69 kDa	2	25	34
**Serpin A3-2**	A2I7M9 [4]	46 kDa	5	14	20
**Serpin A3-2**	A2I7M9 (+1)	46 kDa	0	9	13
**Serpin A3-5**	A2I7N1	46 kDa	0	8	11
**Serpin A3-6**	A2I7N2	46 kDa	0	8	9
**Fetuin**	Q58D62	43 kDa	2	14	16

We extracted and measured the protein mass in the water phase of eluted fractions of 18:1 RhP-L incubated with serum sample. The molecular weight of the proteins was determined using the bovine protein database and compared to the peptides discovered by ESI-LC-MS. W3.5 mL, W7 mL, W7.5 mL, represent the related eluted volume (0.5 mL per fraction) in water phase. Alpha-fetoprotein is abbreviated as AFP.

Table S2. Assignment of molecular ions detected in MALDI-TOP-MS

Molecular adduct	HSPC [DPPC (18:0/16:0)]	HSPC [DSPC (18:0/18:0)]	TF (t)	18:1 RhP	BSA	SM (d18:1/16:0)
$[M - CH3]^-$	746.6 Da	774.6 Da	-	-	-	-
$[M + DHB - H]^-$	914.6 Da	942.6 Da	-	-	-	-
$[M - H]^-$	-	-	866.6 Da	-	-	-
$[M - NH4]^-$	-	-	-	1284 Da	-	-
$[M + H]^+$	-	-	-	-	66 kDa	703.6 Da
$[M + 2H]^{2+}$	-	-	-	-	33 kDa	-
$[M + 3H]^{3+}$	-	-	-	-	22 kDa	-
$[M + Na]^+$	-	-	-	-	-	725.6 Da

Abbreviations: HSPC, L-α-phosphatidylcholine, hydrogenated (Soy) (from manufacturer as a mixture of 11.4% DPPC and 88.6% DSPC); BSA, Bovine serum albumin; SM, Sphingomyelin; 18:1 RhP, 18:1 Rhod PE. HSPC, 18 RhP and TF (t) were detected in negative ion mode, while

BSA and SM were detected in positive ion mode. Monoisotopic masses, except BSA molecular weight.

Table S3: Co-localization coefficient analysis of two labels in DFLP.

Dye	TF (h)	TF (t)	16:0 RhP	18:1 RhP
**Pearsons's**	0.8264 ± 0.02757	0.697 ± 0.03881	0.7901 ± 0.02489	0.7001 ± 0.01628
**Manders' (tM1)**	0.7751 ± 0.06693	0.6461 ± 0.03672	0.8472 ± 0.008945	0.8486 ± 0.02461
**Manders' (tM 2)**	0.8976 ±0.04382	0.6279 ± 0.02704	0.8292 ± 0.01191	0.5778 ± 0.02531

Pearson's: correlation coefficient of TF (h), TF (t), 16:0 RhP and 18:1 RhP with DiD.
Manders': overlap coefficients, tM1 can be defined as the co-occurrence fraction of the counterpart label [TF (h), TF (t), 16:0 RhP or 18:1 RhP] to DiD above threshold, likewise, tM2 is the co-occurrence fraction of DiD with the counterpart label above threshold. Data represents mean ± SEM of at least 15 cells of 3 individual experiments.

Table S4: The excitation and emission wavelengths for detection of fluorophores on Wallac Victor Plate Reader.

Fluorophore	Excitation (nm)	Emission (nm)
TF (t)/TF (h)/ NBD-PE	485	535
DID	615	670
18:1 RhP/16:0 RhP	545	580

Table S5: The excitation and emission wavelengths for detection of fluorophores on spectro-fluorimetry.

Fluorophore	Excitation (nm)	Emission (nm)	Slit (nm)
TF (t)/TF (h)	495	520	5
DiD	644	665	5
NBD-PE	460	535	5
18:1/16:0 RhP	560	583	5

Table S6: Laser power and power in focus spot.

Wavelength (nm)	Laser power intensity	Laser power (mW)	Diameter (μm)	Area (μm^2)	Laser power in focus spot ($mW/\mu m^2$)
**405**	1%	0.01122	0.145	0.016	0.683
**488**	1%	0.01195	0.174	0.024	0.501
**488**	2%	0.02231	0.174	0.024	0.935
**561**	0.5%	0.0277	0.200	0.032	0.879
**561**	1%	0.0559	0.200	0.032	1.773
**633**	4%	0.18	0.226	0.040	4.484
**633**	5%	0.226	0.226	0.040	5.630
**633**	8%	0.364	0.226	0.040	9.068

To measure the laser power in the focus spot, we have used Abbe's law of diffraction to firstly calculate the diameter (d) of the laser spot, which can then be used to calculate the area (A) of the laser spot.

Equation. S1.

$$d = \lambda/(2 \times NA)$$

Where λ is the laser excitation wavelength, and NA is the numerical aperture of the objective. Then the laser power in the focal spot (P_f) can be calculated as following:

Equation. S2.

$$P_f = P/A$$

Where P is the used laser power, and A is the area of the laser spot. The calculated results can be found in **table S6**.

Wavelength and laser power intensity are the laser properties used in described confocal microscopy imaging (in each figure legend), respectively. The laser power in the focus spot is calculated based on equation S1 and equation S2.

Figure S1. Fluorescent liposome (FLPs) characteristics. (a) Size and PdI of a single DiD-labeled liposome, significance is indicated in contrast to a control LP containing 0.005 mol%

DiD. Result implying that inserting additional DiD at a higher concentration (0.05 mol%) may affect the carrier properties. The properties of **(b)** freshly prepared single fluorescent LPs (SLPs) and plain LPs, as well as **(c)** Dual fluorescent LPs (DFLPs) during an 8-week period. *$p<0.05$, **$p<0.01$ for comparison of each subgroup at time zero. **(d)** Toxicity of plain liposomes was determined using BLM, cell viability was expressed as a percentage, and error bars shows SEM, n=3. *$p<0.05$ when compared with control. Statistics were completed using one-way ANOVA with multiple comparisons except for the toxicity assay, which utilized a non-parametric test for comparison of each subgroup at zero.

Figure S2: P**attern of dissociation of each fluorescent lipid nanoparticles (FLPs)**. **(a)** Autofluorescence of elution samples, including serum and plain LPs in serum samples. The orange dashed line indicates the autofluorescence maximum and the yellow curve (serum) is shifted 2 data units for better viewing. **(b)** The representative elution profile for DFLP (18:1 RhP/DiD-L) in serum. The first peak, cutoff point and second peak are denoted as light blue, black and orange dash line, respectively. **(c)** Phosphorus assay of SEC eluent of LPs and 18:1 RhP-L incubated with serum**.** The red and black elution curves are shifted 30 and 70 points for better viewing, respectively. **(d)** Phosphorus assay of SEC eluent of LPs and 18:1 RhP-L incubated with HEPES. The red and black curves are shifted 10 and 40 points for better viewing, respectively.

Figure S3. Mass spectrum of BSA in positive ion mode. (a) The masses at *m/z* 66.6 kDa, 33.3 kDa, and 22.1 kDa, respectively, were considered to be the three charged molecular ions $[M + H]^{+}$, $[M + 2H]^{2+}$, and $[M + 3H]^{3+}$. The red stars denote the positive signal near the red dashed line, while the red dashed lines denote the mass of molecular ion. **(b)** Representative image of the elution profile of 18:1 RhP-L (18:1 Rhodamine PE labeled LP) incubated with serum, data is shown as mean without error bar for better viewing. W3 to W12 corresponded to the volume of eluted samples (0.5 mL per fraction) extracted in the water phase.

Supplemental discussion 1.

For the lipoprotein-related mass

It is important to note that because to the limitation of size-based approaches, we cannot definitively determine whether the lipoprotein-related mass in the first peak is genuinely liposome-bound, or eluted in the first peak because of other reasons.[1] However, given the size differences and high incidence of liposome-protein complexes formed by lipoproteins,[2] we propose that co-existence of lipoproteins in the first SEC peak may be due to lipid exchange from serum to LP[3-5] or lipoprotein adhesion to the LP surface.[4, 6]

Figure S4. Mass spectrum of serum in the positive mode. (a) The two fragment ions at *m/z* 703.6 and 725.6 were identified as sphingomyelin (SM d18:1/16:0) $[M + H]^+$ and SM d18:1/16:0 $[M + Na]^+$, respectively, which were identified as part of LDL and HDL compositions. The red dashed lines denote the mass of the molecular ion, while the red stars denote the positive signal near the red dashed line. **(b)** The elution profile of 18:1 RhP-L treated with serum is representative. C3 to C12 correspond to samples from each elution volume extracted in the chloroform layer.

Figure S5. Dissociation percentage of label from fluorescent liposomes (FPs). (a) Comparison of label dissociation percentage from single FLP (SFLP) and dual FLP (DFLPs) in serum and HEPES. Two-way ANOVA with multiple comparisons was used to complete the statistics. **(b)** Schematic of label detachment from DFLPs in serum. The dissociation percentage **(c)** and elution pattern **(d)** of 18:1 RhP/DiD-L incubated with 100 % serum for 1 h and 24 h were compared. The dissociation percentage **(e)** and elution pattern **(f)** of TF (t)/DiD-L incubated for 24 hours with 10% and 100% serum were compared. The first peak, cutoff point and second peak are denoted as light blue, black and orange dash line, respectively. An unpaired t test was used to analyze these data. The error bars represent the standard error of the mean (SEM), n=3, ***p<0.001, ****p<0.0001, for comparisons within each subgroup.

Figure S6. Schematic illustration of fluorescent lipid extraction and UV light validation. The numbers 1 and 2 correspond to pure TF (t) and pure 18:1 RhP incubated with serum for 24 hours, while the numbers 6 to 24 correspond to 18:1 RhP-L in serum elution volumes from fraction 6 to 24, as well as the retention volumes of 3 to 12 mL. Following lipid extraction, each sample was divided into three layers using the chloroform/methanol/water procedure, with fluorescent lipids appearing in the chloroform phase (bottom layer) and being visible under UV light , while serum salts and protein appeared in the water phase (upper layer).

Figure S7. Pure TF (t) and 18:1 RhP mass spectra observed in negative mode MALDI-TOF-MS. Before lipid extraction, pure TF (t) **(a)** and 18:1 RhP **(b)** were treated with 100 % serum at 37°C for 24 hours. The mass of the pure TF (t) $[M - H]^-$ ion at *m/z* 866 and the mass of the pure 18:1 RhP $[M - NH_4]^-$ ion around *m/z* 1284 (isotope averaged values) were used as standards, as indicated by the red dashed lines. The red stars indicate the presence of a positive signal that is only detectable in the chloroform phase, whereas the black stars indicate the presence of serum noise.

Figure S8. Negative ion mass spectra of SEC eluent of 18:1 RhP-L (18:1 Rhodamine PE labeled LP) in serum. (a) 18:1 RhP-L was incubated in serum for 24 hours and eluted using SEC, followed by extraction and detection of masses (18:1 RhP) in the chloroform layer. Pure serum following extraction was used as a control. The red dashed line indicates the subject of interest. C3 mL to C12 mL are the chloroform phase extracted elution sample. **(b)** The elution profile of 18:1 RhP-L treated with serum is representative. Data is shown as mean without error bar for better viewing.

Figure S9. MALDI-TOF-MS analysis of HSPC in negative mode. In chloroform phase, eluted fractions of 18:1 RhP-L incubated with serum were isolated. **(a)** Mass of HSPC (from manufacturer as a mixture of 11.4% DPPC and 88.6% DSPC), the loss of one methyl group results in the molecular fragments DSPC 18:0/18:0 $[M – CH_3]^-$ and DPPC 18:0/16:0 $[M – CH_3]^-$ at *m/z* 746 and 774, respectively. **(b)** The fragment ions at *m/z* 942 and 914 were classified as

[DSPC 18:0/18:0 + DHB - H]$^-$ and [DPPC 18:0/16:0 + DHB - H]$^-$. This was accomplished by attaching DHB to the HSPC molecule and subsequently releasing a hydrogen atom. Using stock HSPC and serum as positive and noise control, respectively. The red dashed line denotes the area of interest. Peaks nearby the red stars were considered positive signals, while the dark stars denoted serum noise signals. C3 to C12 correspond to the chloroform phase extracted elution samples.

Figure S10. Zoom in on the mass of HSPC at *m/z* 774 and 746.

Figure S11. Mass spectrum study of PEG-DSPE (elution fraction of 18:1 RhP-L in serum) in negative mode. (a) Chloroform phase and **(b)** water phase show, repeating units of 44 Da (the adjacent two clusters are shown by a dashed red line), generated by the loss of one C_2H_4O (ethylene oxide). C3–C12 and W3–W12 corresponded to elution volume 3-12 mL in chloroform and water phase extractions, respectively. The positive signal was observed only in C3 to C5.5 and C7.5, confirming our findings described in the main text.

Figure S12. Lipid mass in water phase by MALDI-TOF-MS negative ion mode. Elution fraction (18:1 RhP-L in serum) was extracted. The **(a)** 18:1 RhP [M – NH_4]$^-$ ion and **(b)** HSPC 36:0 [M – CH_3]$^-$, HSPC 18:0/16:0 [M – CH_3]$^-$ ions were identified in the water phase. The red dashed lines indicate the area of interest. The data revealed that these lipids were not present in the water phase, corroborating our conclusion. W3 mL to W12 mL correspond to extracted solution samples collected from the water layer.

Figure S13. Flow cytometry analysis of FLPs uptake by BLM. (a) Uptake of DFLPs [TF(t)/DiD-L)] by BLM cells, confirm to the results described in the main text. **(b)** Two single FLPs **(**SL-FNPs) TF (t)-L and DiD-L were pre-mixed and incubated with BLM cells for 0.5, 2, 5, and 24 h, respectively. The populations were gated using the positive fluorescence intensities of TF (t) and DiD, and the blue dots represent the LP-free control group. **Merge:** the cumulative plot at all time points. **(c)** Schematic illustration of uptake process of two pre-mixed SFLPs by tumor cells, and the mean fluorescence intensity **(d)** at various time points as determined by flow

cytometry (results reflect the mean standard error of three independent experiments). **(e)** Populations are indicated by the colors red, orange, green, and dark green, which correspond to the incubation period.

Supplemental discussion 2. For co-localization analysis (For figure 7), single particle tracking and photobleaching issues (For figure 8).

In principle, when short-term detailed imaging is performed laser power may be increased and detector gain lowered to improve signal to noise ratio. However, Fluorescence microscopy is accompanied by photobleaching, which is the degradation of fluorophores by (laser) light, which negatively impacts imaging.[7] We observed that photobleaching can be minimized by lowering the laser power, which can be compensated by increased gain, or elongating the time interval when conducting time lapse imaging. Indeed, when NP tracking is most essential a more stably associated probe is preferred when this is possible with respect to the imaging time. When determining co-localization of fluorescent probe, photobleaching has to be taken into account. One may decide to use pixel-based rather than intensity-based analyzes including Manders' Co-localization Coefficients (MCC) for co-localization determination[8] and Spearman for spatiotemporal cross-correlation assessment,[9] to demonstrate the choice of two labels and define the localization/tracking of NPs. Interestingly, we might be able to distinguish the intracellular label dissociation by observing their movement, which coincided with the movement of cellular membrane structures indicated where the free labels [e.g., TF (t)] were attached, while the fluorescent signal moved along with intracellular vesicles (e.g., endo-lysosome system) indicated where NPs [e.g., 16:0 RhP, TF (h)] were encapsulated. However, giving the fact that free dye (e.g., 18:1 RhP) could also be taken up by cells through endo-lysosome pathway, it is difficult to determine whether individual markers are separated from NPs by motion characteristics of a signal, highlighting the complexity of determining nanoparticle localization and NP tracking in cells by individual label, as well as the necessity of dual-labeling strategy.

Figure S14. Time lapse movie of DFLPs incubated with BLM tumor cells after 24 h. (a) TF (t)/DiD-L incubated with BLM. Yellow arrows indicate dissociated TF (t) attached to and move along with intracellular membrane structures. Confocal setting: 63 × 1.4 oil objective; TF (t)

channel: laser 488; 1%; PMT gain, 650; DiD channel: laser 633, 8%; HYD gain 300. **(b)** Representative image of DiD intensity change in time sequence with the same setting as in **a**, except that the laser power of DiD channel is lower, indicating that photobleaching can be diminished by decreasing laser power. Setting: laser 633, 4%; HYD gain, 300. White arrows indicate that the signal in the DiD channel were diminished by laser excitation after short time interval imaging (3.453 s) during 55 s. **(c)** 16:0 RhP/DiD-LPs incubated with BLM. White arrows indicate that the signal in DiD channel diminished less when time interval was extended (5.179 s) during the 57 s sequence. 16:0 RhP channel: laser 561, 0.5%; HYD gain, 200. DiD channel: laser 633, 8%; HYD gain 300;

Figure S15. Live cell imaging for 3 hours of 18:1 RhP/DiD-L uptake by BLM tumor cells. A pre-transfection with LAMP1-GFP to visualize lysosomes was performed for 16 hours (note that the transfection is not homogeneous according to the specification by the manufacture), after which media was withdrawn and cells were cultured with 0.45 mM 18:1 RhP/DiD-L without washing for 3 hours in a confocal microscope. 18:1 RhP has a clear signal localized in lysosomes, which is not aligned with the DiD signal, especially at the earlier incubation time points, as shown by white arrows. Whereas the yellow arrows indicate the colocalized signal between 18:1 RhP and DiD channel. Setting: 63 × 1.4 oil objective, channel for lysosome GFP: laser 488, 2%; PMT gain, 800. 18:1 RhP: laser 561, 0.5 %; HYD gain, 100. DiD channel: 633 nm laser, 5%; HYD gain, 300 (note that 633 laser was kept as a low excitation to avoid quenching because of prolonged imaging). Interval time: 20 minutes, total time:3 hours.

Figure S16. Live cell imaging of 18:1 RhP/DiD-L in HS578T tumor cells. Cells were incubated with 0.45 mM 18:1 RhP/DiD-L without washing for 1.5 h in a confocal microscope. White arrows indicate the obvious signal of 18:1 RhP that is non-aligned with the DiD signal over time. Confocal setting: 63 × 1.4 oil objective, 18:1 RhP channel: laser 561, 1%; HYD gain: 100. DiD channel: laser 633, 5%; HYD gain: 300 (note that laser 633 was kept as a low excitation to avoid quenching because of prolonged imaging). Time interval: 5 mins, total time: 1.5 h.

1. Simonsen, J. B.; Münter, R., *Angewandte Chemie (International ed. in English)* **2020,** *59* (31), 12584-12588. DOI 10.1002/anie.202004611.
2. Onishchenko, N.; Tretiakova, D.; Vodovozova, E., *Acta biomaterialia* **2021,** *134*, 57-78. DOI 10.1016/j.actbio.2021.07.074.
3. Müller, J.; Prozeller, D.; Ghazaryan, A.; Kokkinopoulou, M.; Mailänder, V.; Morsbach, S.; Landfester, K., *Acta biomaterialia* **2018,** *71*, 420-431.
4. Lima, T.; Bernfur, K.; Vilanova, M.; Cedervall, T., *Scientific reports* **2020,** *10* (1), 1-9.
5. Simonsson, C.; Bastiat, G.; Pitorre, M.; Klymchenko, A. S.; Béjaud, J.; Mély, Y.; Benoit, J. P., *European journal of pharmaceutics and biopharmaceutics : official journal of Arbeitsgemeinschaft fur Pharmazeutische Verfahrenstechnik e.V* **2016,** *98*, 47-56. DOI 10.1016/j.ejpb.2015.10.011.
6. Pattipeiluhu, R.; Crielaard, S.; Klein-Schiphorst, I.; Florea, B. I.; Kros, A.; Campbell, F., *ACS central science* **2020,** *6* (4), 535-545.
7. Han, D.; Goudeau, B.; Manojlovic, D.; Jiang, D.; Fang, D.; Sojic, N., *Angewandte Chemie (International ed. in English)* **2021,** *60* (14), 7686-7690. DOI 10.1002/anie.202015030.
8. Manders, E. M. M.; Verbeek, F. J.; Aten, J. A., *Journal of microscopy* **1993,** *169* (3), 375-382. DOI 10.1111/j.1365-2818.1993.tb03313.x.
9. Das, R.; Hammond, S.; Holowka, D.; Baird, B., *Biophysical journal* **2008,** *94* (12), 4996-5008. DOI 10.1529/biophysj.107.105502.
